# AntiAngioPred: A Server for Prediction of Anti-Angiogenic Peptides

**DOI:** 10.1371/journal.pone.0136990

**Published:** 2015-09-03

**Authors:** Azhagiya Singam Ettayapuram Ramaprasad, Sandeep Singh, Raghava Gajendra P. S, Subramanian Venkatesan

**Affiliations:** 1 Chemical Laboratory, Central Leather Research Institute, Council of Scientific and Industrial Research, Adyar, Chennai, India; 2 Bioinformatics Centre, CSIR-Institute of Microbial Technology, Chandigarh, India; University of Rome, ITALY

## Abstract

The process of angiogenesis is a vital step towards the formation of malignant tumors. Anti-angiogenic peptides are therefore promising candidates in the treatment of cancer. In this study, we have collected anti-angiogenic peptides from the literature and analyzed the residue preference in these peptides. Residues like Cys, Pro, Ser, Arg, Trp, Thr and Gly are preferred while Ala, Asp, Ile, Leu, Val and Phe are not preferred in these peptides. There is a positional preference of Ser, Pro, Trp and Cys in the N terminal region and Cys, Gly and Arg in the C terminal region of anti-angiogenic peptides. Motif analysis suggests the motifs “CG-G”, “TC”, “SC”, “SP-S”, etc., which are highly prominent in anti-angiogenic peptides. Based on the primary analysis, we developed prediction models using different machine learning based methods. The maximum accuracy and MCC for amino acid composition based model is 80.9% and 0.62 respectively. The performance of the models on independent dataset is also reasonable. Based on the above study, we have developed a user-friendly web server named “AntiAngioPred” for the prediction of anti-angiogenic peptides. AntiAngioPred web server is freely accessible at http://clri.res.in/subramanian/tools/antiangiopred/index.html (mirror site: http://crdd.osdd.net/raghava/antiangiopred/).

## Introduction

The process of growth of new capillary blood vessels is used for healing and reproduction, which is known as angiogenesis. It occurs for healing wounds and for restoring blood flow to tissues after injury. The control of angiogenesis is achieved by maintaining balance between growth and inhibitory factors in healthy tissues [1, 2]. Angiogenesis is regulated by ‘on’ and ‘off’, switches. Angiogenesis-stimulating growth factors are considered as ‘on switches’ while the angiogenesis inhibitors are considered as the ‘off switches’. Excess production of angiogenic growth factors favors the growth of blood vessels while the presence of excess of angiogenic inhibitors prevents angiogenesis. Recent studies have identified several endogenous anti-angiogenic peptides identified from various biological sources, which regulate angiogenesis and tumor growth [3–6].

There are several peptides derived from various proteins, which inhibit angiogenesis [3, 4, 7–12]. Matrix Metalloproteinase also generates angiogenic inhibitors in vitro by proteolytically cleaving fragments from the pericellular matrix to generate endostatin, tumstatin, angiostatinetc [13]. These peptides inhibit endothelial cell proliferation, migration, tube formation and matrigel neovascularization. For example, the anti-angiogenic properties of arresten are mediated through α1β1 integrin [7]. Recently, novel anti-angiogenic activity was localized to amino acids 54–132 using deletion mutagenesis of tumstatin [9]. The peptides, similar to tumstatin and the tum-5 domain, bind and function via αvβ3 in an RGD-independent manner.

The increasing interest in peptide based therapeutics has led to the development of many peptide databases with therapeutic properties like anticancer [14], antihypertensive [15], antimicrobial [16], blood-brain barrier [17], antiparasitic [18], hemolytic [19], quorum-sensing [20], tumor homing [21] and cell penetrating [22]. So far, peptide based drugs have been employed for many diseases and these are being investigated in clinical applications against tumors, either for imaging or therapy [3, 23–26]. In general, they are attractive molecules as therapeutics because of their natural availability, ability to penetrate cells, specific target binding, and diverse modifications giving flexibility for different applications. The discovery of angiogenesis peptide inhibitors will help in the development of therapeutic treatments against cancer. Several web-based tools are available for the annotation of protein sequence to understand the family and subfamily of the protein [27–29]. So far, there are no web-based tools to predict the anti-angiogenic peptides. Thus, the search of anti-angiogenic agents for the treatment of cancer is particularly important. Hence, in this study, a systematic attempt has been made to develop machine learning based models using various features extracted from peptide sequences like binary profile patterns (BPP); amino acid composition (AAC) as well as dipeptide compositions (DPC). A user-friendly web server has also been developed to help the experimental biologist to predict the anti-angiogenic peptides.

## Methods

### Datasets

#### Positive dataset

The main dataset was collected from the literature. In this study, we have obtained 257 anti-angiogenic peptides from various research articles and patents (S5 Table). Due to the redundancy in the sequences, CD-HIT software was used to eliminate highly similar sequences and it was ensured that no two sequences have more than 70% sequence identity. The resulting dataset contains 135 sequences in the positive dataset (S1 Table). Among these 135 sequences, 20% of the dataset (~28 sequences) was kept separately to be used as independent dataset (S3 Table).

#### Negative dataset

As there is no source of experimentally proven non-anti-angiogenic peptides, we extracted 135 random peptide regions from proteins from Swiss-Prot database [30] and treated them as non-anti-angiogenic peptides (S1 Table). Though some of these randomly selected peptides could be anti-angiogenic in nature but the probability is very less. The random peptide sequences were extracted in such a way that the length distribution of the dataset remains same as of positive dataset. Among these 135 sequences, 20% of the dataset (~28 sequences) was kept separately to be used as independent dataset.

#### Terminus datasets

We divided the main dataset into nine terminus datasets, which are NT5, CT5, NTCT5, NT10, CT10, NTCT10, NT15, CT15 and NTCT15. NT5 and CT5 contain first five residues and last five residues from the N-terminal and C-terminal region of the peptide sequence respectively. NTCT5 is obtained by joining the NT5 and CT5 sequence. Similarly other terminus datasets were also constructed to understand the region of the peptide containing maximum information to discriminate these peptides from random sequence.

#### Independent dataset

The independent dataset was made by extracting 20% of the sequences (~28 sequences) from the positive, as well as negative dataset, thereby making a total of 56 sequences (S2 Table). These sequences were not used in either training or testing procedure while developing any model.

#### Random datasets

In order to check the reliability of models, we created five more random negative dataset using the same procedure as used in developing negative dataset. These datasets have been created to check whether the property of the developed model changes if the negative dataset is replaced with another randomly created dataset. These datasets were named as ‘Random1’, ‘Random2’, ‘Random3’, ‘Random4’ and ‘Random5’ (S4 Table).

#### Calculation of residue propensities

The propensity of each amino acid in anti-angiogenic peptides was calculated by the following formula:

$$P\left(i\right)=\frac{AACp\left(i\right)}{AACp\left(i\right)+AACs\left(i\right)}$$ (Eq 1)

where, *P(i)* represents propensity of *i* ^*th*^ amino acid, AAC*p(i)* and *AACs(i)* represents the average composition of *i* ^*th*^ amino acid in positive and Swiss-Prot dataset, respectively. We also calculated the position wise propensities of amino acids in both N-terminal and C-terminal regions of the peptides.

#### Cross validation technique

In the present study, we performed ten-fold cross-validation technique to develop our models. In this technique, the sequences were randomly divided into ten sets. Nine sets were used for training the model while the remaining tenth set was used for testing. The process was then repeated ten times such that each set was once used as a test set. The average performance of all the ten sets is reported as the final performance of the method.

#### Machine learning approaches

Different machine learning techniques like Support Vector Machines (SVM), Neural Networks (Multilayer Perceptron), Bayesian approach (Naïve Bayes) [31], Nearest Neighbor (IBk) [32], Decision trees (Random Forest and J48) [33, 34] and logistic regression [35] were used to develop the models. SVM based method was implemented using SVM^Light^ software [36] while rest of the methods were implemented using WEKA package [37].

#### Input features for prediction

A machine learning based method requires set of features in the form of numbers as input. These features contain the global information of the biological molecules being studied by the method. The features used in this study are described below.

#### Amino acid composition (ACC)

It is represented by the percentage of each amino acid within a peptide with a vector size of 20. It was calculated by using the following equation:

$$ACC\left(i\right)=\frac{A_{i}}{N}X10$$ (Eq 2)

Where *AAC(i)* represent the percentage of amino acid (i); *A*_*i*_ represent the frequency of i^th^ residue and *N* is the total number of residues in the peptide.

#### Dipeptide composition

Dipeptide composition refers to the percentage of all the possible pair of amino acids (e.g. AA, AC, AD etc.) present in the peptide. It represents a vector size of 400 (20 x 20) and also includes information about the neighboring residues. It was calculated using the following equation:

$$DPC\left(i\right)=\frac{DP\left(i\right)}{N}$$ (Eq 3)

Where *DPC (i)* represents the percentage of dipeptide (i); *DP (i)* represents the frequency of *i* ^*th*^ dipeptide and *N* represents the total number of dipeptides.

#### Binary profile

In binary profile, each amino acid is represented by a binary vector of size 20 where one element of the vector corresponding to the presence of a particular amino acid is represented by 1 and other 19 elements are represented by 0. (e.g. Ala by 1,0,0,0,0,0,0,0,0,0,0,0,0,0,0,0,0,0,0,0). Therefore for a stretch of 5 amino acids, the total vector size of binary profile will be 100 (20 x 5).

#### Two sample logos

Online service of two sample logo software was used to generate two sample logos [38–40]. It is useful in representing the frequency of amino acids at specific positions in the peptide sequence. The size of the residues displayed at each position is proportional to the relative frequency of each amino acid at that position.

#### Performance measures

The performance of the developed models was calculated using the standard performance parameters like Sensitivity (Sn), Specificity (Sp), Accuracy (Acc) and Matthew’s correlation coefficient (MCC). The formula to calculate Sensitivity, Specificity, Accuracy and MCC is given by following equations:

$$Sensitivity=\frac{TP}{\left(TP+FN\right)}$$ (Eq 4)

$$Specificity=\frac{TN}{\left(TN+FP\right)}$$ (Eq 5)

$$Accuracy=\frac{\left(TP+TN\right)}{\left(TP+FP+TN+FN\right)}X\;100$$ (Eq 6)

$$MCC=\frac{\left(TP\right)\left(TN\right)-\left(FP\right)\left(FN\right)}{\sqrt{\left(TP+FN\right)\left(TN+FP\right)\left(TP+FP\right)\left(TN+FN\right)}}$$ (Eq 7)

Where TP, TN, FP and FN represents True Positive, True Negative, False Positive and False Negative respectively.

## Results

### Amino acid composition analysis

**Fig 1:**
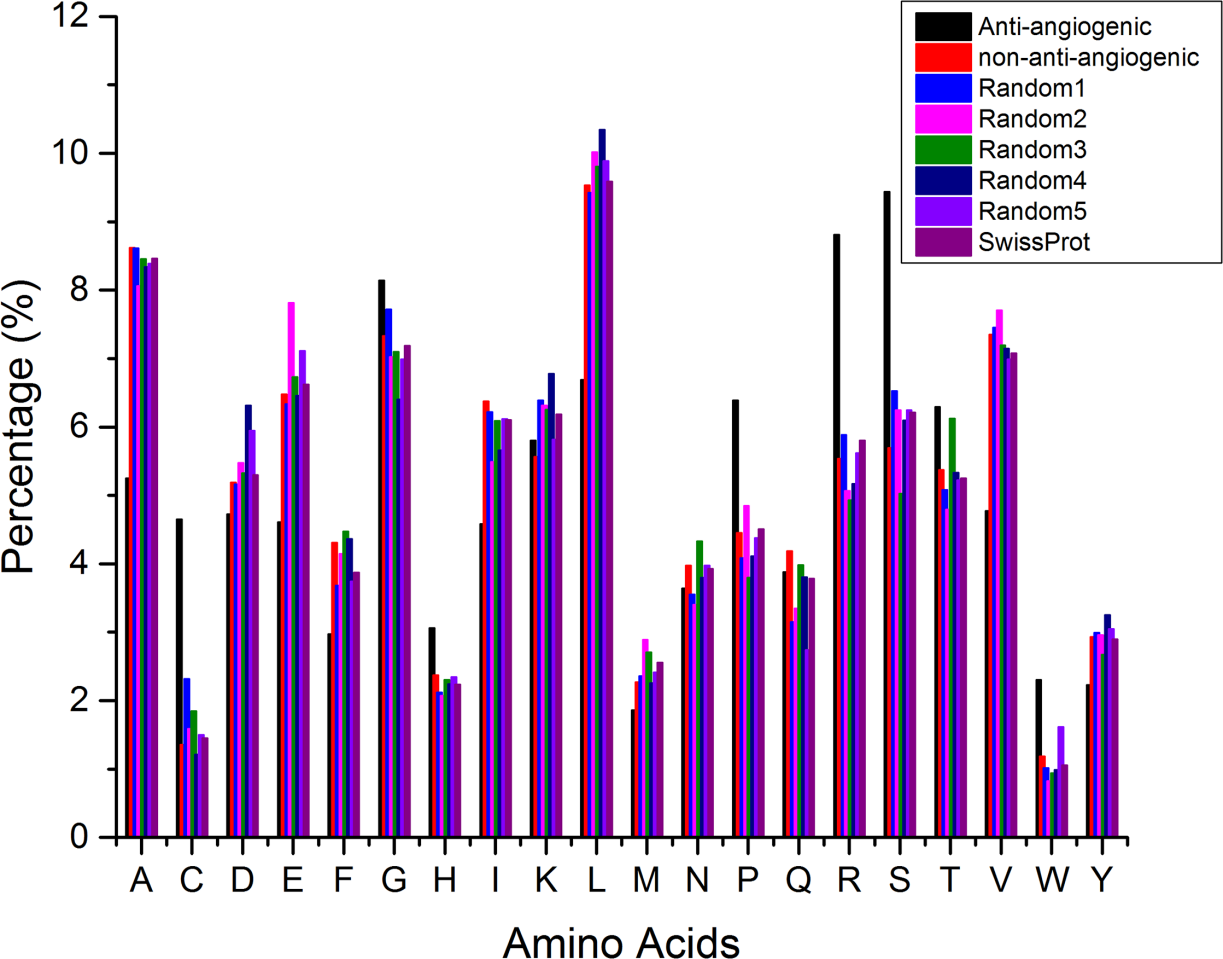
Amino acid composition analysis of the residues in anti-angiogenic and non-anti-angiogenic peptides. Composition of entire Swiss-Prot is taken for reference and composition of all random datasets.

The amino acid composition analysis was carried out to extract certain residues, which are dominant in anti-angiogenic peptides. We compared the average amino acid composition in anti-angiogenic and non-anti-angiogenic peptides (Fig 1). It was observed that residues like Cys, Pro, Ser, Arg, Trp, Thr and Gly are predominant in anti-angiogenic peptides while residues Ala, Asp, Ile, Leu, Val and Phe are under-represented in these peptides. Composition was also computed for all the random datasets and compared with the negative dataset and no bias was observed, ensuring that the dataset is purely random. We also calculated the average amino acid composition of the entire Swiss-Prot database to be used as reference for analyzing the difference (Fig 1).

### Residue propensities and positional preference

The propensities of residues are in accordance with the amino acid composition analysis with Cys, Trp, Ser, Arg and Pro being predominant in anti-angiogenic peptides while Val, Ala, Leu and Ile being less preferred in these peptides (S6 Table). To understand the position wise preference of amino acids at the first and last 10 residues of the N and C terminus of anti-angiogenic peptides, we calculated the position wise propensities using Swiss-Prot as reference dataset (S7 Table). Cys, Ser, Thr and His are preferred at N1 position; Pro at N2 position; Trp and Pro at N3; Ser and Phe at N4 and Cys is predominant at N5, N6 and N7 positions in anti-angiogenic peptides. At C-terminal region, Cys is prominent at C1 and C2; Gly and Cys at C3 and C4; Cys at C5 while Arg is most favoured at C8, C9 and C10 position. We also performed residue based preference analysis using two sample logo (S1 and S2 Fig), which is in accordance with the results described above.

### Motif analysis

To find the frequent motifs in the anti-angiogenic peptides, we extracted the motifs using MERCI software using following criteria: i) the motif should be present in at least 10% (~14 peptides) of the total number of peptides in the positive dataset, ii) the motif can have a maximum of 5 gaps. Here, the gap represents the presence or absence of any amino acid. Using the above criteria, we obtained a total of 151 motifs. Further, we selected the motifs, which had propensity (Eq 1) more than or equal to 0.90. This resulted in a total of 22motifs, which are "CG-G", "TC", "SC", "SP-S", "W-S-C", "WS-C", "S-T-C", "S-C-S", "CS-T", "C-S-T", "T-C", "S-C", "C-G-G", "TR", "S-T-G", "S-P-S", "SP", "RT", "P-W", "P-C", "C-N" and "CG" (hyphen ‘-’ represents a gap).These motifs are important for understanding and identification of anti-angiogenic peptides. The full list of 151 motifs sorted by propensity is given in S8 Table.

### Performance of various machine learning approaches on the dataset

We used different machine learning classifiers like SVM, Random Forest (RF), IBk, J48, Naïve Bayes, Logistic and Multilayer Perceptron (MP) to develop amino acid composition based model on whole peptide dataset. This helps us to compare the performance of different classifiers on the same dataset. The models developed in this study are explained below.

### Amino acid composition based model

We used amino acid composition of the peptide as input feature to develop the prediction model using SVM, J48, RF, Naïvebayes, MP, Logistic and IBk machine learning classifiers (Table 1).SVM (MCC = 0.48), MP (MCC = 0.49) and RF(MCC = 0.48) based models performed better than other methods. The performance among the best models (SVM, RF, MP) was alike and therefore we selected SVM machine learning method for further development of models using different input features.

**Table 1:** Performance of various machine learning classifiers using amino acid composition as input feature on whole peptide dataset.

Methods	Sn (%)	Sp (%)	Acc (%)	MCC
SVM	69.2	78.5	73.8	0.48
IBk	69.2	74.8	72.0	0.44
Random Forest	70.1	77.6	73.8	0.48
Logistic	70.1	74.8	72.4	0.45
Multilayer Perceptron	70.1	78.5	74.3	0.49
Naïve Bayes	65.4	72.0	68.7	0.37
J48	62.6	73.8	68.2	0.37

**Sn:** Sensitivity; **Sp:** Specificity; **Acc:** Accuracy; **MCC:** Matthew’s Correlation Coefficient.

The performance of SVM based models developed on the terminus datasets is summarized in Table 2. We observed that the best results in terms of accuracy (80.9%) and MCC (0.62) are obtained on NT15 terminus dataset. The results on CT5 terminus dataset had least accuracy (65.6%) and MCC (0.31). The results indicate that the major functional properties of these peptides are contained in the N-terminal residues of the peptide sequence.

**Table 2:** Performance of SVM based models using amino acid composition as input feature on nine terminus datasets.

Approach	Sn (%)	Sp (%)	Acc (%)	MCC
NT5	69.2	72.0	70.6	0.41
CT5	62.9	68.2	65.6	0.31
NTCT5	72.0	71.0	71.5	0.43
NT10	71.0	76.6	73.8	0.48
CT10	69.8	70.1	70.0	0.40
NTCT10	68.2	76.6	72.4	0.45
NT15	79.0	82.7	80.9	0.62
CT15	67.9	72.8	70.4	0.41
NTCT15	75.3	80.3	77.8	0.56

**Sn:** Sensitivity; **Sp:** Specificity; **Acc:** Accuracy; **MCC:** Matthew’s Correlation Coefficient.

### Dipeptide composition based model

SVM based model was developed using dipeptide composition of the whole peptide as input feature and we achieved an accuracy of 74.8% with MCC 0.50 (Table 3). We also developed models on nine terminus datasets as done previously. The maximum accuracy (75.9%) and MCC (0.52) was obtained on CT15 terminus dataset although the performance on NT15 (74.1% accuracy) was also nearby. There was a slight increase in the performance of dipeptide composition based model (74.8% accuracy) as compared to amino acid composition based model (73.8% accuracy) on whole peptide dataset.

**Table 3:** Performance of SVM based models using dipeptide composition as input feature on whole peptide dataset and nine terminus datasets.

Approach	Sn(%)	Sp (%)	Acc (%)	MCC
Whole peptide	75.7	73.8	74.8	0.50
NT5	66.4	66.4	66.4	0.33
CT5	57.1	57.9	57.6	0.15
NTCT5	64.5	60.8	62.6	0.25
NT10	70.1	73.8	72.0	0.44
CT10	63.2	70.1	66.7	0.33
NTCT10	65.4	74.8	70.1	0.40
NT15	75.3	72.8	74.1	0.48
CT15	74.1	77.8	75.9	0.52
NTCT15	72.8	76.5	74.7	0.49

**Sn:** Sensitivity; **Sp:** Specificity; **Acc:** Accuracy; **MCC:** Matthew’s Correlation Coefficient.

### Binary profile based model

We also developed SVM based models using binary profile of peptide as input feature. We achieved the best accuracy (77.6%) and MCC (0.55) on NTCT5 terminus dataset (Table 4). Binary profile based models performed poor on both NT15 and CT15 terminus datasets with MCC of 0.43 and 0.41 respectively.

**Table 4:** Performance of SVM based models using binary profile as input feature on nine terminus datasets.

Approach	Sn(%)	Sp (%)	Acc (%)	MCC
NT5	66.4	72.9	69.7	0.39
CT5	68.6	69.2	68.9	0.38
NTCT5	75.7	79.4	77.6	0.55
NT10	65.4	72.0	68.7	0.37
CT10	55.7	61.7	58.7	0.17
NTCT10	65.4	71.0	68.2	0.37
NT15	70.4	72.8	71.6	0.43
CT15	69.1	71.6	70.4	0.41
NTCT15	71.6	76.5	74.1	0.48

**Sn:** Sensitivity; **Sp:** Specificity; **Acc:** Accuracy; **MCC:** Matthew’s Correlation Coefficient.

### Performance on independent dataset

In order to validate the models, the performances of all the best models were tested on an independent dataset. The amino acid and dipeptide composition based models, both achieved accuracy 69.6% with MCC 0.41 on whole peptide dataset (Table 5). The model based on amino acid composition on NT15 terminus dataset achieved accuracy 75.0% with MCC 0.51. These results indicate that our models are robust and performed equally well on the independent dataset.

**Table 5:** Performance of SVM based models on independent dataset.

Approach	Feature	Sn (%)	Sp (%)	Acc (%)	MCC
Independent Dataset					
Whole peptide	AAC	53.6	85.7	69.6	0.41
Whole peptide	DPC	64.3	75.0	69.6	0.41
NT15	AAC	65.0	85.0	75.0	0.51

**Sn:** Sensitivity; **Sp:** Specificity; **Acc:** Accuracy; **MCC:** Matthew’s Correlation Coefficient.

### Reliability of models

We created five random datasets (Random-1–5) and developed amino acid composition based model using positive dataset and each of the random datasets generating a total of 5 models. The performance of these models is given in Table 6. The results indicate that the developed models are reliable and stable enough to perform well in all the random datasets.

**Table 6:** Performance of SVM based models using amino acid composition as input feature on different random dataset taken as negative dataset.

Dataset	Sn (%)	Sp (%)	Acc (%)	MCC
Random Datasets				
Random1	70.1	71.8	71.0	0.42
Random2	70.1	79.4	74.8	0.50
Random3	75.7	77.6	76.6	0.53
Random4	75.7	81.3	78.5	0.57
Random5	72.9	73.8	73.4	0.47

**Sn:** Sensitivity; **Sp:** Specificity; **Acc:** Accuracy; **MCC:** Matthew’s Correlation Coefficient.

### Implementation of web server

Based on the above study and to assist the scientific community, we developed a web server named ‘AntiAngiopred’ with user-friendly interface. We implemented two models in the web server; i) amino acid composition based model on N15 terminus dataset, ii) amino acid composition based model on whole peptide dataset. The former model is implemented due to its best performance among other models and the latter is implemented for peptides which are less than 15 residues in length. Due to the limited number of anti-angiogenic peptide sequences, the models implemented in the web server are developed using all the sequences. A user can submit the peptide sequence in the ‘Predict’ module of the web server and can predict whether his/her peptide has anti-angiogenic property or not. User can also get the single mutant analogs of the submitted peptide along with their prediction. It will also help a user to identify minimum mutations and their location in a peptide sequence so as to have anti-angiogenic properties in that peptide. If a user has multiple peptides then ‘Multiple Peptide’ module helps him/her to predict the anti-angiogenic nature of all of his/her peptides using a single submission form. The web service can be accessed at http://clri.res.in/subramanian/tools/antiangiopred/ or at its mirror site at http://crdd.osdd.net/raghava/antiangiopred/

## Discussion

In this study an attempt has been made to develop an effective in silico method to predict anti-angiogenic peptides. We used a dataset of 107 positive and 107 negative sequences to develop models and check performance of models using ten-fold cross validation technique. We also tested the performance of the developed models on the independent dataset with 28 positive and 28 negative sequences. Primary analysis based on the amino acid composition and residue propensities reveal that the residues such as Cys, Trp, Ser, Arg and Pro are preferred in anti-angiogenic peptides while Val, Ala, Ile and Asp are not preferred in these peptides. Analysis of two sample logos and positional preference show that the predominance of certain residues like Ser, Pro, Trp and Cys in the N-terminal region of anti-angiogenic peptides, while in the C-terminus, the residues such as Cys, Gly and Arg were found. Both Ser and Cys have high propensities while Ala and Val have low propensities at most of the positions in the N-terminal region. In C-terminal region, Arg, His and Cys have high propensities while Ala has low propensity at most of the positions. Further, motif analysis suggests the prominent motifs like "CG-G", "TC", "SC", "SP-S", "W-S-C", "WS-C", etc., which are present in the anti-angiogenic peptides. Based on the primary analysis, we developed models for discriminating anti-angiogenic and non-anti-angiogenic peptides using different machine learning techniques. The SVM based models developed in this study, were able to discriminate anti-angiogenic and non-anti-angiogenic peptides with 80.9% accuracy and 0.62 MCC on NT15 dataset using amino acid composition as input feature. On an independent dataset, the above model achieved 75% accuracy and 0.51 MCC. Further, the performance of amino acid composition based models on whole peptide dataset developed using all the random datasets indicate that the model is stable and hence reliable. To assist and help the scientific community, we have integrated the models developed in this study in the web server AntiAngioPred, which can be accessed at http://clri.res.in/subramanian/tools/antiangiopred/index.html (mirror site: http://crdd.osdd.net/raghava/antiangiopred/)

### Limitations and future development

The current study is based on the small size of the dataset of anti-angiogenic peptides. Therefore, the predictor may not be robust enough to apply on a very diverse set of peptides as compared to the dataset used in this study. As soon as more and more anti-angiogenic peptides will be made available in the literature, the predictor will require retraining on the new dataset to make it more robust. The choice of random peptides as negative dataset poses a further limitation on this predictor. Ideally, a negative dataset should have experimentally validated non anti-angiogenic peptides. However, in the absence of non anti-angiogenic peptides, a more appropriate choice would be random peptides having similar physico-chemical properties as that of anti-angiogenic peptides. The above suggestions should be considered for the future development of models.

## Supporting Information

S1 Fig Two sample logo of first 10 residues of N-terminal region of the anti-angiogenic and non-anti-angiogenic peptides representing positional preference of amino acids. (DOCX)

S2 Fig Two sample logo of last 10 residues of C-terminal region of the anti-angiogenic and non-anti-angiogenic peptides representing positional preference of amino acids. (DOCX)

S1 Table Positive and Negative Datasets (135 sequences each). (DOCX)

S2 Table Dataset used for 10 fold cross validation. (DOCX)

S3 Table Independent Dataset (28 positive and 28 negative sequences). (DOCX)

S4 Table Random Datasets used in this study. (DOCX)

S5 Table All (257) anti-angiogenic peptides extracted from literature (literature reference is given in header line of the fasta formatted peptide sequences). (DOCX)

S6 Table Propensities of amino acids in anti-angiogenic peptides calculated using Swiss-Prot as reference dataset. (DOCX)

S7 Table Positional propensity of amino acids in first and last 10 residues of N- and C- terminus of anti-angiogenic peptides. N1 represents the first residue and C10 represents the last residue. The propensities were calculated using Swiss-Prot as reference dataset.
(DOCX)

S8 Table List of motifs extracted by MERCI software. (DOCX)

## References

[1] Brem S, Cotran R, Folkman J. Tumor angiogenesis: a quantitative method for histologic grading. Journal of the National Cancer Institute. 1972;48(2):347–56. . 4347034

[2] Folkman J. Anti-angiogenesis: new concept for therapy of solid tumors. Annals of surgery. 1972;175(3):409–16. 10.1097/00000658-197203000-00014 . 5077799 PMC1355186

[3] Rosca EV, Koskimaki JE, Rivera CG, Pandey NB, Tamiz AP, Popel AS. Anti-angiogenic peptides for cancer therapeutics. Curr Pharm Biotechnol. 2011;12(8):1101–16. 21470139 10.2174/138920111796117300 PMC3114256

[4] Koskimaki JE, Karagiannis ED, Rosca EV, Vesuna F, Winnard PT Jr., Raman V, et al Peptides derived from type IV collagen, CXC chemokines, and thrombospondin-1 domain-containing proteins inhibit neovascularization and suppress tumor growth in MDA-MB-231 breast cancer xenografts. Neoplasia. 2009;11(12):1285–91. 20019836 10.1593/neo.09620 PMC2794509

[5] Sulochana KN, Ge R. Developing antiangiogenic peptide drugs for angiogenesis-related diseases. Curr Pharm Des. 2007;13(20):2074–86. Epub 2007/07/14. . 17627540 10.2174/138161207781039715

[6] Karagiannis ED, Popel AS. A systematic methodology for proteome-wide identification of peptides inhibiting the proliferation and migration of endothelial cells. Proc Natl Acad Sci U S A. 2008;105(37):13775–80. 10.1073/pnas.0803241105 18780781 PMC2544530

[7] Nyberg P, Xie L, Sugimoto H, Colorado P, Sund M, Holthaus K, et al Characterization of the anti-angiogenic properties of arresten, an alpha 1 beta 1 integrin-dependent collagen-derived tumor suppressor. Experimental Cell Research. 2008;314(18):3292–305. 10.1016/j.yexcr.2008.08.011 . 18775695 PMC2613512

[8] Maeshima Y, Manfredi M, Reimer C, Holthaus KA, Hopfer H, Chandamuri BR, et al Identification of the anti-angiogenic site within vascular basement membrane-derived tumstatin. Journal of Biological Chemistry. 2001;276(18):15240–8. 10.1074/jbc.M007764200 . 11278365

[9] Maeshima Y, Colorado PC, Kalluri R. Two RGD-independent alpha(v)beta(3) integrin binding sites on tumstatin regulate distinct anti-tumor properties. Journal of Biological Chemistry. 2000;275(31):23745–50. 10.1074/jbc.C000186200 . 10837460

[10] Kohn EC. Endostatin and angiostatin: the next anti-angiogenesis generation. Angiogenesis. 1998;2(1):25–7. 10.1023/a:1009046208807 . 14517372

[11] Tolsma SS, Volpert OV, Good DJ, Frazier WA, Polverini PJ, Bouck N. PEPTIDES DERIVED FROM 2 SEPARATE DOMAINS OF THE MATRIX PROTEIN THROMBOSPONDIN-1 HAVE ANTI-ANGIOGENIC ACTIVITY. Journal of Cell Biology. 1993;122(2):497–511. 10.1083/jcb.122.2.497 . 7686555 PMC2119646

[12] Osborne S, Horwell DC, Howson W, inventors; Warner Lambert Co, assignee. New peptide analogues acting as NK-2 receptor antagonists|are useful as analgesics, anti-angiogenic agents for treating e.g. rheumatoid arthritis or tumours, for appetite suppression or treating psychosis patent US5554644-A.

[13] Stetler-Stevenson WG. Matrix metalloproteinases in angiogenesis: a moving target for therapeutic intervention. J Clin Invest. 1999;103(9):1237–41. Epub 1999/05/04. 10.1172/JCI6870 10225966 PMC408361

[14] Tyagi A, Tuknait A, Anand P, Gupta S, Sharma M, Mathur D, et al CancerPPD: a database of anticancer peptides and proteins. Nucleic Acids Res. 2015;43(Database issue):D837–43. Epub 2014/10/02. 10.1093/nar/gku892 gku892 [pii]. . 25270878 PMC4384006

[15] Kumar R, Chaudhary K, Sharma M, Nagpal G, Chauhan JS, Singh S, et al AHTPDB: a comprehensive platform for analysis and presentation of antihypertensive peptides. Nucleic Acids Res. 2015;43(Database issue):D956–62. Epub 2014/11/14. 10.1093/nar/gku1141 gku1141 [pii]. . 25392419 PMC4383949

[16] Waghu FH, Gopi L, Barai RS, Ramteke P, Nizami B, Idicula-Thomas S. CAMP: Collection of sequences and structures of antimicrobial peptides. Nucleic Acids Research. 2014;42(D1):D1154–D8. 10.1093/nar/gkt1157 . 24265220 PMC3964954

[17] Van Dorpe S, Bronselaer A, Nielandt J, Stalmans S, Wynendaele E, Audenaert K, et al Brainpeps: the blood-brain barrier peptide database. Brain Structure & Function. 2012;217(3):687–718. 10.1007/s00429-011-0375-0 . 22205159

[18] Mehta D, Anand P, Kumar V, Joshi A, Mathur D, Singh S, et al ParaPep: a web resource for experimentally validated antiparasitic peptide sequences and their structures . Database: the journal of biological databases and curation. 2014;2014 10.1093/database/bau051 . PMC4054663 24923818

[19] Gautam A, Chaudhary K, Singh S, Joshi A, Anand P, Tuknait A, et al Hemolytik: a database of experimentally determined hemolytic and non-hemolytic peptides. Nucleic Acids Research. 2014;42(D1):D444–D9. 10.1093/nar/gkt1008 . 24174543 PMC3964980

[20] Wynendaele E, Bronselaer A, Nielandt J, D'Hondt M, Stalmans S, Bracke N, et al Quorumpeps database: chemical space, microbial origin and functionality of quorum sensing peptides. Nucleic Acids Research. 2013;41(D1):D655–D9. 10.1093/nar/gks1137 . 23180797 PMC3531179

[21] Kapoor P, Singh H, Gautam A, Chaudhary K, Kumar R, Raghava GPS. TumorHoPe: A Database of Tumor Homing Peptides . Plos One. 2012;7(4). 10.1371/journal.pone.0035187 . PMC3327652 22523575

[22] Gautam A, Singh H, Tyagi A, Chaudhary K, Kumar R, Kapoor P, et al CPPsite: a curated database of cell penetrating peptides . Database: the journal of biological databases and curation. 2012;2012:bas015-bas. 10.1093/database/bas015 . PMC3296953 22403286

[23] Xu Y, Jiang YF, Wu B. New agonist- and antagonist-based treatment approaches for advanced prostate cancer. J Int Med Res. 2012;40(4):1217–26. Epub 2012/09/14. . 22971474 10.1177/147323001204000401

[24] Oka Y, Tsuboi A, Fujiki F, Shirakata T, Nishida S, Hosen N, et al "Cancer antigen WT1 protein-derived peptide"-based treatment of cancer-toward the further development. Curr Med Chem. 2008;15(29):3052–61. Epub 2008/12/17. . 19075652 10.2174/092986708786848631

[25] Pilla L, Rivoltini L, Patuzzo R, Marrari A, Valdagni R, Parmiani G. Multipeptide vaccination in cancer patients. Expert Opin Biol Ther. 2009;9(8):1043–55. Epub 2009/07/14. 10.1517/14712590903085109 . 19591629

[26] Thundimadathil J. Cancer treatment using peptides: current therapies and future prospects. J Amino Acids. 2012;2012:967347 Epub 2013/01/15. 10.1155/2012/967347 23316341 PMC3539351

[27] Thomas PD, Campbell MJ, Kejariwal A, Mi HY, Karlak B, Daverman R, et al PANTHER: A library of protein families and subfamilies indexed by function. Genome Research. 2003;13(9):2129–41. 10.1101/gr.772403 . 12952881 PMC403709

[28] Wu CH, Huang HZ, Yeh LSL, Barker WC. Protein family classification and functional annotation. Computational Biology and Chemistry. 2003;27(1):37–47. 10.1016/s1476-9271(02)00098-1 . 12798038

[29] Hunter S, Jones P, Mitchell A, Apweiler R, Attwood TK, Bateman A, et al InterPro in 2011: new developments in the family and domain prediction database. Nucleic Acids Res. 2012;40(Database issue):D306–12. Epub 2011/11/19. 10.1093/nar/gkr948 gkr948 [pii]. 22096229 PMC3245097

[30] Apweiler R BA, Martin MJ, O'Donovan C, Magrane M, Alam-Faruque Y, Alpi E, Antunes R, Arganiska J, Barrera Casanova E, Bely B, Bingley M, Bonilla C, Britto R, Bursteinas B, Mun Chan W, Chavali G, Cibrian-Uhalte E, Da Silva A, De Giorgi M, Fazzini F, Gane P, Castro LG, Garmiri P, Hatton-Ellis E, Hieta R, Huntley R, Legge D, Liu W, Luo J, MacDougall A, Mutowo P, Nightingale A, Orchard S, Pichler K, Poggioli D, Pundir S, Pureza L, Qi G, Rosanoff S, Sawford T, Shypitsyna A, Turner E, Volynkin V, Wardell T, Watkins X, Zellner H, Corbett M, Donnelly M, van Rensburg P, Goujon M, McWilliam H, Lopez R, Xenarios I, Bougueleret L, Bridge A, Poux S, Redaschi N, Aimo L, Auchincloss A, Axelsen K, Bansal P, Baratin D, Binz PA, Blatter MC, Boeckmann B, Bolleman J, Boutet E, Breuza L, Casal-Casas C, de Castro E, Cerutti L, Coudert E, Cuche B, Doche M, Dornevil D, Duvaud S, Estreicher A, Famiglietti L, Feuermann M, Gasteiger E, Gehant S, Gerritsen V, Gos A, Gruaz-Gumowski N, Hinz U, Hulo C, James J, Jungo F, Keller G, Lara V, Lemercier P, Lew J, Lieberherr D, Lombardot T, Martin X, Masson P, Morgat A, Neto T, Paesano S, Pedruzzi I, Pilbout S, Pozzato M, Pruess M, Rivoire C, Roechert B, Schneider M, Sigrist C, Sonesson K, Staehli S, Stutz A, Sundaram S, Tognolli M, Verbregue L, Veuthey AL, Wu CH, Arighi CN, Arminski L, Chen C, Chen Y, Garavelli JS, Huang H, Laiho K, McGarvey P, Natale DA, Suzek BE, Vinayaka C, Wang Q, Wang Y, Yeh LS, Yerramalla MS, Zhang J. Activities at the Universal Protein Resource (UniProt). Nucleic Acids Res. 2014;42(Database issue):D191–8. Epub 2013/11/21. 10.1093/nar/gkt1140 gkt1140 [pii]. 24253303 PMC3965022

[31] Langley. Estimating Continuous Distributions in Bayesian Classifiers. Eleventh Conference on Uncertainty in Artificial Intelligence,; San Mateo1995. p. 338–45.

[32] Daad K. Instance-based learning algorithms: Springer; 1991 37–66 p.

[33] L B. Random Forests Machine Learning2001.

[34] Quinlan R. Programs for Machine Learning. San Mateo, CA: Morgan Kaufmann Publishers,; 1993.

[35] le Cessie SavH JC. Ridge Estimators in Logistic Regression. Applied Statistics. 1992;41:191.

[36] Scholkopf B BC, Smola A, editor. Making large-scale SVM learning practical: Cambridge, MA: MIIT Press; 1999.

[37] Mark Hall EF, Geoffrey Holmes, Bernhard Pfahringer, Peter Reutemann, Witten Ian H . The WEKA data mining software: An update . SIGKDD Explorations. 2009;11(1).

[38] Vacic V, Iakoucheva LM, Radivojac P. Two Sample Logo: a graphical representation of the differences between two sets of sequence alignments. Bioinformatics. 2006;22(12):1536–7. Epub 2006/04/25. btl151 [pii] 10.1093/bioinformatics/btl151 . 16632492

[39] Crooks GE, Hon G, Chandonia JM, Brenner SE. WebLogo: a sequence logo generator. Genome Res. 2004;14(6):1188–90. Epub 2004/06/03. 10.1101/gr.849004 14/6/1188 [pii]. 15173120 PMC419797

[40] Schneider TD, Stephens RM. Sequence logos: a new way to display consensus sequences. Nucleic Acids Res. 1990;18(20):6097–100. Epub 1990/10/25. 2172928 10.1093/nar/18.20.6097 PMC332411

