# Supplementary material for: AntiAngioPred: A Server for Prediction of Anti-Angiogenic Peptides
Source: PLoS One. 2015 Sep 3;10(9):e0136990. doi: 10.1371/journal.pone.0136990 (PMC4559406; doi:10.1371/journal.pone.0136990)
Supplement: S1 Fig — (DOCX) [file pone.0136990.s001.docx]

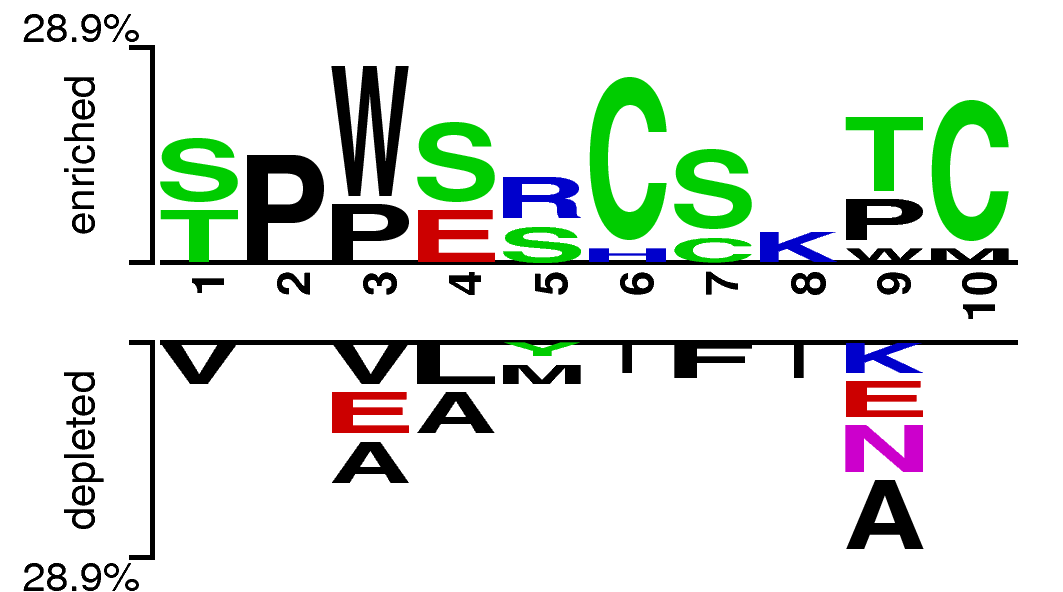


S1 Fig: Two sample logo of first 10 residues of N-terminal region of the anti-angiogenic and non-anti-angiogenic peptides representing positional preference of amino acids.
