# Supplementary material for: AntiAngioPred: A Server for Prediction of Anti-Angiogenic Peptides
Source: PLoS One. 2015 Sep 3;10(9):e0136990. doi: 10.1371/journal.pone.0136990 (PMC4559406; doi:10.1371/journal.pone.0136990)
Supplement: S1 Table — (DOCX) [file pone.0136990.s003.docx]

**S1 Table** Positive and Negative Datasets (135 sequences each)

| Positive Dataset (135 sequences) | Negative Dataset (135 sequences) |
| --- | --- |
| AAPFLECQGRQGTCHFFAN | ADNWQSFDRWKDH |
| ANIKLSVQMKLFKRHLKWKIIVKLNDGRELSLDA | AEALAALRALADKNQVF |
| ARPAKAAATQKKVERKAPDA | AERWREAAKLI |
| ASWSACSVSCGGGARQRTR | AFAQFGSDLDAATQKLLNRGARLTELMKQPQ |
| ATPFIECSGARGTCHYFAN | AGAGYALLALIGTEAAS |
| CDSDSDITWDQLWDLMK | AKAAETKSSSEQELRITQS |
| CELDENNTPMC | ALVIGVIYATSMIFQSTSLV |
| CETWRTETTGATGQASSLLSGRLLEQKAASCHNSYIVLCIENSFMTSFSK | ATSINNSSLPDV |
| CKITRCPMIPCYISSPDECLWMDWVTEKNINGHQAKFFACIKRSDGSCAWYRGAAPPKQEFLDIEDP | AVVQKRFGFPEGSV |
| CQNHHAKHGKVC | AVYLFYGTKDCL |
| DDDDDNDKIPDDRDN | CSRDNKHTLHRE |
| DDDDKRAGSPSGGPFCALARQPLTGSPPNERAFFCSSRDV | DDVWNMKYLRGFKWADLMEQVQRE |
| DFKLFAVYIKYR | DIAPDTLENLISEFVLREG |
| DGRELCLDPKENWVQRVVEKFLK | DKAFIAFLEETFDQFLP |
| DGRKICLDPDAPRIKKIVQKKL | DLDDESIQGKLNFENFSLL |
| DLWIRETLTSPKSLTG | DMEAFTKLTDNIFLE |
| DPFFKVPVNKLAAAVSNFGYDLYRVRSSTSPTTN | DVSKLKEGEQYMSFCTFPGHSALM |
| DPPEGLUGTKPPROH | EALDAARYYANV |
| DRSTREPIYMSTI | EASGPSFVSSHYLQESPGGISLEGSELTFPD |
| DSSPVSTEQLAPTA | EDWSLDSRPGKSTKNSRNK |
| DTAVTGLASPLSTGKILDQKAYSCANRLIVLCIENSFMTDARK | EGELLILENVRFNKGEKKDD |
| EDMNQKLFDLRGKFKRPPLRRVRMSADAML | EHNDLRLCCKQIVEEA |
| EGLPGPQGPKGFPGLPGLTG | EKAGLKIVAAKMLQLSQAQAEG |
| EIPSCESSASPDQSDSSVPPEE | EKQIEQLVAQDLVRHFADLYRIDIPT |
| EKSSRPEFYKVILGAHEEYIRG | ELLIEDHIKTACNWGTTHK |
| EKYEGKISKTMSGLDCQAWDS | ENAKNRLGLAQAD |
| ESLARPCAPGAPAEARL | ENGSTAIVVGRPITQAADPQKAYE |
| FCNINNVCNFASRNDYSYW | ERYAALLHDLGKAKTPSDILPRHHGHDLAGVEPVRKVNQRLRAPKHCAEL |
| FLKDHRISTFKNWPF | EYIDGSVIAQL |
| FLSSRLQDLYSIVRRADRAA | FEPQVMKIMANVRPDRQTVLFSATFPRNMEALARKTLNKPVEIVVGGKSVVAPEITQIVEVR |
| GDVIDTDRDIDR | FFTPSASHPAYVNFA |
| GFHDHGPCDPPSHK | FGRLGTMFGSDLYNIKPDLV |
| GHRATSDLASTGEESQD | FTSALSRAQKT |
| GPWEDCSVSCGGGEQLRSR | FTVRKISNGEGVERAFQTH |
| GPWEPCSVTCSKGTRTRRR | GALTDPTAQLVYLQKDGGL |
| GPWERCTAQCGGGIQARRR | GGPAERLTYEGDYNARGV |
| GPWGDCSRTCGGGVQFSSR | GHENISTTQIYTHLDFQHLADVYDQAHPRARKKSSQHKEE |
| GPWGPCSGSCGPGRRLRRR | GIVFQFFNLIPTLTVLENITLP |
| GVDITVIRPNH | GKGVKTEFNRHVEDIKRESDGAWVL |
| GYCSWYRGWAPPDKSIINATDP | GKSVADAIAILTFTPNKAAEII |
| HGLGHGHEQQHGLGHGHKFKLDDDLEHQGGHVLD | GLHKGNKVNLTLRPAPANTGLIFRRVD |
| HGSTTLRDITV | GMIFLELNFKGAEEIYYKHVHCRGGCSVFFSKISGVLTFM |
| HHPHGHHPHGHHPHGHHPHG | GPEGMLSIAAPARDLKLATIELEHSHPLGRLWDIDVLTPEGEILSRRDYSLPPRRCLLCEQSAAVCA |
| HKLINTEGHHS | GRLKGEELAQYNLWLDYLDALE |
| HNRTPENFPCKNL | GSGRTDARVHAQGQ |
| HTHQDFQPVLHLVALNTPLSGGMRGIR | GSKFDSSLDRNRPFEFTLGAGQVIK |
| IMRIKQGQIGQMTI | GTSIVGIVENGISVLGKIF |
| INEFLERSGIPRQRNQ | HGGRVTLMEITDDGLAILQFGGGCNGCSMVDFTL |
| INGSLDKRLLPDVET | HSGNIWVDSDPARKSNPRFIVLD |
| INGSLDKRVQDCYHG | HSSREKIVIPFFSLLIKDIYFLNEGCA |
| INLEACLGRTLMD | HVLSRLSYISALGMMTRITS |
| INLEACLKRGRT | HWMYQGKHVLIIFDD |
| ITMQGIQGQKIRMIMF | IAELGTAEFPRLRIGIGRPAP |
| IYSFDGRDIMTDPSWPQKVIWHGSSPHGVRLVDNYCEAWRTA | ICRDIDLVRKLIKQAGLSLLAVERQENFPD |
| KAFDITYVRLKF | IEHPVLMARKPRFR |
| KCGHKHQCAVHN | IEVTHWVQSRRAYAQGALEAARRLIGRPP |
| KIKSCYYLPCFVTS | IFTFAGLIDHSHDFIIGFHAV |
| KNECLWTDMLSNFGYPGYQSKHYACIRQKG | IHRAAGPALINACY |
| KRFKQDGGWSHWSPWSSCSVTCGDGVITRIRLCNSPSPQMNGKPCEGEARETKACKKDACPI | IIYITEEMGLLLGYSPIEILEKRF |
| KSVRGKGKGQKRKRKKSRYK | INLTIAVHNGR |
| LHCPALVTYNTDTFESMPNPEGRYTFGASCV | IVDDWIYMIEEICKI |
| LLRISLLLIQSWLE | IWIDPGFGFAKSVQQNTELLKGLDRVCQLGYPVL |
| LPGLTGSKGVRGISGLPGFSG | KEVDAKYIETKRSIVQHITQIPYY |
| LRRFSTMPFMFCNINNVCNF | KFGADCKYKFES |
| LRSRGELVAKFLAGEQSPEDYVAE | KLCGTNSDAYGFSANLDDS |
| LSSTCILVLVKDILVLVVKEILVLVVKDKPI | KLLDIADLHSEMKPLH |
| LVPLPKIKNSTFT | KMVTADYIKEGA |
| LVPRGSRAGSPSGGPFCALARQPLTGARLMSGLFFALHET | KQVKDKVPDGVFIFLTPPDLAELKSRIIGR |
| MEPECNLNCTD | KTSTEAGVNLVVG |
| MFSPILSLEIILALATLQSVFAQPVICTTVGSAAEGS | KVSVWSKVLRSDAAWDDK |
| MLQNSAVLLLLVISASA | LASAYGLAKHRDGRWEWA |
| MPTWAWWLFLVLLLALWAPARG | LGYLGPDLADSAIAVNESIIPKFLRLVDPTAAELQNF |
| NGKQVCLDPEAPFLKKVIQKILDS | LHRGRIPEHQREESEV |
| NGREACLDPEAPMVQKIVQKMLKG | LLSEYIPSVPNCWSLLKNKKT |
| NGRKACLNPASPIVKKIIEKMLNS | LMEYEQNENPMK |
| NGRKISLDLRAPLYKKIIKKLLES | LVAPVTVGKGA |
| NVLLSPLSVATALSALSLGAEQRTES | LVVVPPYVIRY |
| PGLKGKRGDSGSPATWTTRG | MASGNAVCGSSAIAAVEP |
| PTGERLRTCERLSYP | MQSLVDIAAVTELAHAAGAKV |
| QEPHRHSIFTPQTNPRADLEKN | MRIVDLGAAPGGWSQVAAKK |
| QMIVIELGTNPLKSSGIENGAFQGMK | MTGLVKWFNPE |
| QPWGTCSESCGKGTQTRAR | MVFITVSTGVGGGVVSGGKLLTGPGG |
| QPWSQCSATCGDGVRERRR | MVSSEKAMANPDSMEIDSQTISQQVLITSQSGSV |
| QQMNQKDFLSLIVS | MYNSLLRMTGACHKKCVPPH |
| QRTESIIHRALYYDLIS | NDNTPEILYPTI |
| RCRLAERRQIAK | NGWLHCPADPDLIF |
| RGFTKMPHVQIHTEASESL | NITVMTSGFAFHYYVNNPH |
| RIFGESVSLRVQDWEW | NTKFDELMEFP |
| RPFVEMYSEIPE | PDEVTIGIVRERLG |
| RQVFQVAYIIIKA | PDLCSWEEAQLSS |
| RRPAAAGKRRREKQRPSDKPRR | PKLTALVENVAEQQGINLTS |
| RRPKGRAMRREKQRPSDKPRR | PLIVLKDSIGREVINRSLIRVR |
| RRPKGRGKRRREKQRPTDCHLCGDAVPRR | QAGADISMIGQFGVGFYSA |
| SAPFIECHGRGTCNYYANS | QAITDIHLDRV |
| SAWRACSVTCGKGIQKRSR | QAQQKIILETFILFEDEVGKKL |
| SEWSDCSVTCGKGMRTRQR | QGCKMNNINVVYTPWANLKK |
| SKRKSRPVSVKTFEDIPLEEP | QGGAQRGGFTGPIP |
| SKWSECSRTCGGGVKFQER | QLPLQQQQQQQQQQQQQQQ |
| SPNITVTLKKFPL | QMLEEGLLDEVQALLAAGIKGN |
| SPSTHPNEGLEENYCRNPDN | QTTIHVLPTAPTTVNVT |
| SPWDIASVTAGGVQKRS | RAGSKRWLGKRPVVRGVVMNPVDHPHGGGEGRAPIGRKKPTTP |
| SPWSKCSAACGQTGVQTRTR | RELAAEVGSLLT |
| SPWSPCSGNCSTGKQQRTR | RFRVLPQGLKVKQVEREDAGVYVCKATNGFGSLSVNYTLVVL |
| SPWSPCSTSCGLGVSTRI | RHPDCKIVRRRGRV |
| SPWSQCSVRCGRGQRSRQVR | RKEAKRRYNEGALPGFDPA |
| SPWSQCTASCGGGVQTR | RLQLGKMLNLIDESKFA |
| SPWSSASVTAGDGVDITRIR | RPGTPLFTVKAYL |
| SPWTKCSATCGGGHYMRTR | RSERLAKLNQILRI |
| SRTVRKTSRLWSSLSLNTCNNVHSKS | RVEQPENPMLDARVQAFRIA |
| SSTSPHRPRFS | RVIVVFHCEFSSERGPRMCR |
| SVSGGGHHHHHHGGG | SGNMLAGGGTLYLYALGMG |
| TAWGPCSTTCGLGMATRV | SMGPMPESGQLVFQTANLT |
| TEENRELVSELKRP | STDVSWEELRDTE |
| TEWSACNVRCGRGWQKRSR | SYDLGERKPSSAAYQKAPT |
| TEWSVCNSRCGRGYQKRTR | SYRDKEMSATFRQIL |
| TEWTACSKSCGMGFSTRV | TEGIDAMGEVTIRLRRDGQLFSGHAA |
| TGASSEEEDPF | TIASMPAVDEINRLSN |
| TKPPRKRPPKTKKRPPKTTKPPRGZOG | TLPHQRLIVATDRGIFYKM |
| TKWTPCSRTCGMGISNRV | TVEIVMGLEEEFQISVE |
| TLPFAYCNIHQVCHYAQRNDRSYWL | VAATDGVGTKLKIAIDTGN |
| TMPFLFCNVNDCNFASRNDYSYWL | VAFKPNSTNIHVENVTVYG |
| TQWTSCSKTCNSGTQSRHR | VCGTIYVGGKEVNQCMDKTSDNAI |
| TSLDASIIWAMMQN | VCHGNCPQSNNAFFQPLDP |
| TSWSPCSASCGGGHYQRTR | VFSTTSLVVVAHYKGLTVA |
| TSWSQCSKTCGTGISTRV | VIVCLLGTAGLFLPPWLA |
| TTITGKKCQSWAAMFPHRHSKT | VKVIEAVRARTPKTT |
| VGSGGCMFGNGK | VMFAALIFKKDTFFR |
| VIFEWTLLQVLSESDQDQSLEVFLT | VQDFGTALKVPK |
| VVGSPSAQDEASPL | VQWILSFPRAPMGSVSVHV |
| WDLVVVSAGVAEVGV | VRYHYINKAYEVTMKIQIIS |
| WTRCSSSCGRGVSVRSR | VVRLAREPGKRESRYMH |
| YCNINEVCHYARRNDKSYWL | YEDLRDESLKGLVDIGF |
| YPYDVPDYASL | YFLIQSVSSTVMLLNGLYIFVN |
| YRIPIVRRLQRR | YGEPGMQLFVYGREE |
| YTMNPRKLFDY | YNLSDTIKAFSILLLTDLCI |
