# Supplementary material for: AntiAngioPred: A Server for Prediction of Anti-Angiogenic Peptides
Source: PLoS One. 2015 Sep 3;10(9):e0136990. doi: 10.1371/journal.pone.0136990 (PMC4559406; doi:10.1371/journal.pone.0136990)
Supplement: S2 Table — (DOCX) [file pone.0136990.s004.docx]

**S2 Table** Dataset used for 10 fold cross validation

| Positive (107 peptides) | Negative (107 peptides) |
| --- | --- |
| AAPFLECQGRQGTCHFFAN | ADNWQSFDRWKDH |
| ANIKLSVQMKLFKRHLKWKIIVKLNDGRELSLDA | AEALAALRALADKNQVF |
| CDSDSDITWDQLWDLMK | AFAQFGSDLDAATQKLLNRGARLTELMKQPQ |
| CETWRTETTGATGQASSLLSGRLLEQKAASCHNSYIVLCIENSFMTSFSK | AGAGYALLALIGTEAAS |
| CKITRCPMIPCYISSPDECLWMDWVTEKNINGHQAKFFACIKRSDGSCAWYRGAAPPKQEFLDIEDP | AKAAETKSSSEQELRITQS |
| CQNHHAKHGKVC | ATSINNSSLPDV |
| DDDDDNDKIPDDRDN | AVVQKRFGFPEGSV |
| DDDDKRAGSPSGGPFCALARQPLTGSPPNERAFFCSSRDV | DDVWNMKYLRGFKWADLMEQVQRE |
| DGRELCLDPKENWVQRVVEKFLK | DKAFIAFLEETFDQFLP |
| DGRKICLDPDAPRIKKIVQKKL | DMEAFTKLTDNIFLE |
| DLWIRETLTSPKSLTG | DVSKLKEGEQYMSFCTFPGHSALM |
| DPFFKVPVNKLAAAVSNFGYDLYRVRSSTSPTTN | EALDAARYYANV |
| DPPEGLUGTKPPROH | EASGPSFVSSHYLQESPGGISLEGSELTFPD |
| DRSTREPIYMSTI | EDWSLDSRPGKSTKNSRNK |
| DSSPVSTEQLAPTA | EGELLILENVRFNKGEKKDD |
| EGLPGPQGPKGFPGLPGLTG | EHNDLRLCCKQIVEEA |
| EIPSCESSASPDQSDSSVPPEE | EKAGLKIVAAKMLQLSQAQAEG |
| EKSSRPEFYKVILGAHEEYIRG | EKQIEQLVAQDLVRHFADLYRIDIPT |
| EKYEGKISKTMSGLDCQAWDS | ELLIEDHIKTACNWGTTHK |
| ESLARPCAPGAPAEARL | ENGSTAIVVGRPITQAADPQKAYE |
| FCNINNVCNFASRNDYSYW | ERYAALLHDLGKAKTPSDILPRHHGHDLAGVEPVRKVNQRLRAPKHCAEL |
| FLKDHRISTFKNWPF | FEPQVMKIMANVRPDRQTVLFSATFPRNMEALARKTLNKPVEIVVGGKSVVAPEITQIVEVR |
| FLSSRLQDLYSIVRRADRAA | FFTPSASHPAYVNFA |
| GFHDHGPCDPPSHK | FGRLGTMFGSDLYNIKPDLV |
| GHRATSDLASTGEESQD | FTSALSRAQKT |
| GPWEDCSVSCGGGEQLRSR | FTVRKISNGEGVERAFQTH |
| GPWEPCSVTCSKGTRTRRR | GALTDPTAQLVYLQKDGGL |
| GPWERCTAQCGGGIQARRR | GGPAERLTYEGDYNARGV |
| GPWGDCSRTCGGGVQFSSR | GHENISTTQIYTHLDFQHLADVYDQAHPRARKKSSQHKEE |
| GPWGPCSGSCGPGRRLRRR | GIVFQFFNLIPTLTVLENITLP |
| HGLGHGHEQQHGLGHGHKFKLDDDLEHQGGHVLD | GKGVKTEFNRHVEDIKRESDGAWVL |
| HGSTTLRDITV | GKSVADAIAILTFTPNKAAEII |
| HHPHGHHPHGHHPHGHHPHG | GLHKGNKVNLTLRPAPANTGLIFRRVD |
| HKLINTEGHHS | GMIFLELNFKGAEEIYYKHVHCRGGCSVFFSKISGVLTFM |
| HTHQDFQPVLHLVALNTPLSGGMRGIR | GPEGMLSIAAPARDLKLATIELEHSHPLGRLWDIDVLTPEGEILSRRDYSLPPRRCLLCEQSAAVCA |
| IMRIKQGQIGQMTI | GSGRTDARVHAQGQ |
| INEFLERSGIPRQRNQ | GSKFDSSLDRNRPFEFTLGAGQVIK |
| INGSLDKRLLPDVET | GTSIVGIVENGISVLGKIF |
| INGSLDKRVQDCYHG | HGGRVTLMEITDDGLAILQFGGGCNGCSMVDFTL |
| INLEACLKRGRT | HSGNIWVDSDPARKSNPRFIVLD |
| ITMQGIQGQKIRMIMF | HSSREKIVIPFFSLLIKDIYFLNEGCA |
| KAFDITYVRLKF | HVLSRLSYISALGMMTRITS |
| KCGHKHQCAVHN | HWMYQGKHVLIIFDD |
| KIKSCYYLPCFVTS | IAELGTAEFPRLRIGIGRPAP |
| KRFKQDGGWSHWSPWSSCSVTCGDGVITRIRLCNSPSPQMNGKPCEGEARETKACKKDACPI | IEHPVLMARKPRFR |
| KSVRGKGKGQKRKRKKSRYK | IEVTHWVQSRRAYAQGALEAARRLIGRPP |
| LHCPALVTYNTDTFESMPNPEGRYTFGASCV | IFTFAGLIDHSHDFIIGFHAV |
| LLRISLLLIQSWLE | IHRAAGPALINACY |
| LPGLTGSKGVRGISGLPGFSG | IIYITEEMGLLLGYSPIEILEKRF |
| LRRFSTMPFMFCNINNVCNF | INLTIAVHNGR |
| LRSRGELVAKFLAGEQSPEDYVAE | IVDDWIYMIEEICKI |
| LSSTCILVLVKDILVLVVKEILVLVVKDKPI | IWIDPGFGFAKSVQQNTELLKGLDRVCQLGYPVL |
| LVPLPKIKNSTFT | KFGADCKYKFES |
| LVPRGSRAGSPSGGPFCALARQPLTGARLMSGLFFALHET | KLCGTNSDAYGFSANLDDS |
| MEPECNLNCTD | KLLDIADLHSEMKPLH |
| MFSPILSLEIILALATLQSVFAQPVICTTVGSAAEGS | KMVTADYIKEGA |
| MLQNSAVLLLLVISASA | KVSVWSKVLRSDAAWDDK |
| NGKQVCLDPEAPFLKKVIQKILDS | LASAYGLAKHRDGRWEWA |
| NGREACLDPEAPMVQKIVQKMLKG | LGYLGPDLADSAIAVNESIIPKFLRLVDPTAAELQNF |
| NGRKACLNPASPIVKKIIEKMLNS | LHRGRIPEHQREESEV |
| NVLLSPLSVATALSALSLGAEQRTES | LLSEYIPSVPNCWSLLKNKKT |
| PGLKGKRGDSGSPATWTTRG | LMEYEQNENPMK |
| PTGERLRTCERLSYP | LVAPVTVGKGA |
| QEPHRHSIFTPQTNPRADLEKN | LVVVPPYVIRY |
| QMIVIELGTNPLKSSGIENGAFQGMK | MASGNAVCGSSAIAAVEP |
| QPWGTCSESCGKGTQTRAR | MQSLVDIAAVTELAHAAGAKV |
| QPWSQCSATCGDGVRERRR | MRIVDLGAAPGGWSQVAAKK |
| RCRLAERRQIAK | MTGLVKWFNPE |
| RIFGESVSLRVQDWEW | MVFITVSTGVGGGVVSGGKLLTGPGG |
| RPFVEMYSEIPE | MVSSEKAMANPDSMEIDSQTISQQVLITSQSGSV |
| RQVFQVAYIIIKA | MYNSLLRMTGACHKKCVPPH |
| RRPAAAGKRRREKQRPSDKPRR | NDNTPEILYPTI |
| RRPKGRAMRREKQRPSDKPRR | NGWLHCPADPDLIF |
| RRPKGRGKRRREKQRPTDCHLCGDAVPRR | NITVMTSGFAFHYYVNNPH |
| SAWRACSVTCGKGIQKRSR | NTKFDELMEFP |
| SEWSDCSVTCGKGMRTRQR | PDLCSWEEAQLSS |
| SKRKSRPVSVKTFEDIPLEEP | PKLTALVENVAEQQGINLTS |
| SKWSECSRTCGGGVKFQER | QAGADISMIGQFGVGFYSA |
| SPNITVTLKKFPL | QAITDIHLDRV |
| SPSTHPNEGLEENYCRNPDN | QAQQKIILETFILFEDEVGKKL |
| SPWSKCSAACGQTGVQTRTR | QGCKMNNINVVYTPWANLKK |
| SPWSPCSGNCSTGKQQRTR | QMLEEGLLDEVQALLAAGIKGN |
| SPWSPCSTSCGLGVSTRI | QTTIHVLPTAPTTVNVT |
| SPWSQCTASCGGGVQTR | RELAAEVGSLLT |
| SPWTKCSATCGGGHYMRTR | RHPDCKIVRRRGRV |
| SRTVRKTSRLWSSLSLNTCNNVHSKS | RPGTPLFTVKAYL |
| SSTSPHRPRFS | RSERLAKLNQILRI |
| TAWGPCSTTCGLGMATRV | RVEQPENPMLDARVQAFRIA |
| TEWSACNVRCGRGWQKRSR | SGNMLAGGGTLYLYALGMG |
| TEWSVCNSRCGRGYQKRTR | SMGPMPESGQLVFQTANLT |
| TEWTACSKSCGMGFSTRV | STDVSWEELRDTE |
| TGASSEEEDPF | SYDLGERKPSSAAYQKAPT |
| TKPPRKRPPKTKKRPPKTTKPPRGZOG | SYRDKEMSATFRQIL |
| TKWTPCSRTCGMGISNRV | TEGIDAMGEVTIRLRRDGQLFSGHAA |
| TLPFAYCNIHQVCHYAQRNDRSYWL | TIASMPAVDEINRLSN |
| TQWTSCSKTCNSGTQSRHR | TLPHQRLIVATDRGIFYKM |
| TSLDASIIWAMMQN | VAATDGVGTKLKIAIDTGN |
| TSWSQCSKTCGTGISTRV | VAFKPNSTNIHVENVTVYG |
| TTITGKKCQSWAAMFPHRHSKT | VCHGNCPQSNNAFFQPLDP |
| VGSGGCMFGNGK | VFSTTSLVVVAHYKGLTVA |
| VIFEWTLLQVLSESDQDQSLEVFLT | VIVCLLGTAGLFLPPWLA |
| VVGSPSAQDEASPL | VKVIEAVRARTPKTT |
| WTRCSSSCGRGVSVRSR | VQDFGTALKVPK |
| YCNINEVCHYARRNDKSYWL | VVRLAREPGKRESRYMH |
| YPYDVPDYASL | YEDLRDESLKGLVDIGF |
| YRIPIVRRLQRR | YFLIQSVSSTVMLLNGLYIFVN |
| YTMNPRKLFDY | YNLSDTIKAFSILLLTDLCI |
