# Supplementary material for: AntiAngioPred: A Server for Prediction of Anti-Angiogenic Peptides
Source: PLoS One. 2015 Sep 3;10(9):e0136990. doi: 10.1371/journal.pone.0136990 (PMC4559406; doi:10.1371/journal.pone.0136990)
Supplement: S3 Table — (DOCX) [file pone.0136990.s005.docx]

**S3 Table Independent Dataset (28 positive and 28 negative sequences)**

| Positive (28 sequences) | Negative (28 sequences) |
| --- | --- |
| ARPAKAAATQKKVERKAPDA | AERWREAAKLI |
| ASWSACSVSCGGGARQRTR | ALVIGVIYATSMIFQSTSLV |
| ATPFIECSGARGTCHYFAN | AVYLFYGTKDCL |
| CELDENNTPMC | CSRDNKHTLHRE |
| DFKLFAVYIKYR | DIAPDTLENLISEFVLREG |
| DTAVTGLASPLSTGKILDQKAYSCANRLIVLCIENSFMTDARK | DLDDESIQGKLNFENFSLL |
| EDMNQKLFDLRGKFKRPPLRRVRMSADAML | ENAKNRLGLAQAD |
| GDVIDTDRDIDR | EYIDGSVIAQL |
| GVDITVIRPNH | GRLKGEELAQYNLWLDYLDALE |
| GYCSWYRGWAPPDKSIINATDP | ICRDIDLVRKLIKQAGLSLLAVERQENFPD |
| HNRTPENFPCKNL | KEVDAKYIETKRSIVQHITQIPYY |
| INLEACLGRTLMD | KQVKDKVPDGVFIFLTPPDLAELKSRIIGR |
| IYSFDGRDIMTDPSWPQKVIWHGSSPHGVRLVDNYCEAWRTA | KTSTEAGVNLVVG |
| KNECLWTDMLSNFGYPGYQSKHYACIRQKG | PDEVTIGIVRERLG |
| MPTWAWWLFLVLLLALWAPARG | PLIVLKDSIGREVINRSLIRVR |
| NGRKISLDLRAPLYKKIIKKLLES | QGGAQRGGFTGPIP |
| QQMNQKDFLSLIVS | QLPLQQQQQQQQQQQQQQQ |
| QRTESIIHRALYYDLIS | RAGSKRWLGKRPVVRGVVMNPVDHPHGGGEGRAPIGRKKPTTP |
| RGFTKMPHVQIHTEASESL | RFRVLPQGLKVKQVEREDAGVYVCKATNGFGSLSVNYTLVVL |
| SAPFIECHGRGTCNYYANS | RKEAKRRYNEGALPGFDPA |
| SPWDIASVTAGGVQKRS | RLQLGKMLNLIDESKFA |
| SPWSQCSVRCGRGQRSRQVR | RVIVVFHCEFSSERGPRMCR |
| SPWSSASVTAGDGVDITRIR | TVEIVMGLEEEFQISVE |
| SVSGGGHHHHHHGGG | VCGTIYVGGKEVNQCMDKTSDNAI |
| TEENRELVSELKRP | VMFAALIFKKDTFFR |
| TMPFLFCNVNDCNFASRNDYSYWL | VQWILSFPRAPMGSVSVHV |
| TSWSPCSASCGGGHYQRTR | VRYHYINKAYEVTMKIQIIS |
| WDLVVVSAGVAEVGV | YGEPGMQLFVYGREE |
