# Supplementary material for: AntiAngioPred: A Server for Prediction of Anti-Angiogenic Peptides
Source: PLoS One. 2015 Sep 3;10(9):e0136990. doi: 10.1371/journal.pone.0136990 (PMC4559406; doi:10.1371/journal.pone.0136990)
Supplement: S4 Table — (DOCX) [file pone.0136990.s006.docx]

**S4 Table** Random Datasets used in this study

| **Random1** | **Random2** | **Random3** | **Random4** | **Random5** |
| --- | --- | --- | --- | --- |
| AAKIERGSNEPNKKKVGTITTAQL | AFVMQVNPDAHEEYQRRHNPIWPELEAVLKSHGAHNYAIYLDKARNLLFA | AAAKDLNIEALITNSYLEVE | AAACERERSDEEYYAKEVYKIDMPSF | AALLIEINRFFPDALTFPF |
| AAPVLSTLWLGALAALLIE | AGEPDFPTPEP | AALSAVQACRR | AAFSNVSTAEELLEEKYIA | AAQVAAEDAGRKAREHGMETLEIE |
| AFEIPLVAKKLAKSKKY | AGQLIEVNLDLVLGNDITTPVAINE | ACPYDMLKLTD | AAGIEYSKRAAREADIIFCVFD | ACPTGALKIEDLVV |
| AIKRLMDLGCYRGLRHRRGLPLRGQRTRTNARTR | AGQVVTGNPTPHFLSVCRPNYTALGCPPP | AEHDVSILTAQN | AATIALNGLGMDATRVRLLVDPATRRNTHRLQVC | ADSPYAGGVFFLSIHFPTDY |
| ALNGLGMDATRVRLLV | AKFGLKEINPEGEVFNPEFH | AGILYPVPFFIILSVLNTVAIT | AEHLNVFDIVKYN | AEHLVQFYSGFNVFSYLTFRAIVSLLTALFLSLWMGPRLIAWL |
| ALRLEGNTVGVEAARVIAKALEKKSELKR | ALKVFVAGRNRLENDGATALAEAFRVIGTLEEVH | AITTNGAHPSKCVTIQRTLDGRLQVAGRKGFPHVIYARLWRWPDLHKNELKHVKYCQYAFDLKCDSV | AGTAIRESMEIIQANN | AEHVCPEVAVINGGDGR |
| AMKQLIGPGGQKWANMDPEERMLA | ANEKLKAALKARDIVAGIASEEED | AKDYIKSHYVSSFSIYSSLVLGLIIA | AKGFRDAQNPPSSFTLG | AHNYAIYLDKARNLLFAMVEIES |
| APHFVGGGVAFGPRPKVYNLSMNK | APEEHPVLLTEAP | AKIAQLEAALLSSESKVKEQQD | AKIFGNGLEVICRYRAVG | AIDHDNYLEFQQ |
| AQFGLRMQNATGQLGKPSE | ARKGIITPEMEYVALRESL | ALQVTVDAVSGSAKEKIT | ALLDTLLRRYVESQV | ALIEEAKAKATEEGSKIIA |
| ARAGVVKTMSGGRAQSIGRR | ASASLLISNNSSTASSPAP | ALREIEEGLINNQILDVRE | ANAGLVIVTHDTQLASRA | ALSAEQVMAIADLDNRET |
| ASKVKQDMPPVG | ASLRLSEEELLDKLEIF | APSVSALTPHAG | DALCVIAAPVAGLRALCRA | ARQAAKANSHNSALRSKFR |
| ASMFTGKIGYEY | AVVGTSSLRRKAQ | APTMSLECVNVSN | DGVHKIEFEHGTTSGKRVV | AVEKNIIDKKGSWFNYQDRKLGQGR |
| ATPQGRNDRYCESMMRRRG | AVYDGHGGSRVA | ATGQLGKPSELKRVRRDIAR | DHESNTVMFVMQKGYE | AVRRVNVSNPNAPMLTDYIDPKKYTY |
| AVRHDTLLWARDAALVAELAARRENARYLGGVALPPGLRYEADLATALSHAQADDALCVIAAPVAGL | AWENDKPTVVALREIASGLVDENV | AVDLRAIGPRTVTL | DIDADGILHVSAKDKNSGKEQ | AYAVDTSDLAVTA |
| CAVEMMHAGASRYDLDRFGVV | CAIKNGLSLTS | CPPRPEMLIDAIL | DKAKAAEIFLA | DAIILASAGLIRLELVERITQFIPVE |
| CPAFVKWLEEFTTQYSRDQ | DDMIKRALDFRESREAEPHPLW | CSLDKLAQEFELLPTDSGAVV | DNIAYMKNQLKMLG | DLAWICFRESGKHWMVSALDAEKRAIQRFSE |
| DAIDLDVELGPAKPASEEGLVWDEGKCDFIG | DEAGELLEVIKHTTNNVTPPPEACTTWKAMYNGINELIDDLM | DATVLTEYRGLSVVETTELRRA | DRMETEAIKRVFGGHANSMSISSTKSMHAHCLGAASALEMIACVMAIQEG | DPGGGIRLGPLA |
| DCFDDVPEALMAMFGTPQL | DKPITKKPAETRM | DGVITVEEAKGINDDLEVVEGMQFDRGYLS | DRPALAAVFPTEKRTAAILLD | DRTETPPPLASLNVSKLAS |
| DEKKKVIKTWSRRSTIFP | DLKPAEGARRER | DHTLYGEEVKFGGGKV | DSCSECGSFEM | DTVDQAIERAGTKAGNKGF |
| DLRAIGPRTVTLDC | DYFIRIVYTVRYAPNILLH | DLQKSKLTESLKIDLHIAR | EDAVRTLRLARE | DYEHHHHDHEDHHDHGHHHHHEHG |
| DNDIIGTSIYNVTPNKAGKYFVKIHC | EALNAVYRQGRNGEAIGP | DRRKKKSEFRKLWITRINAAVR | EDVERFIREFP | EALFAPAPGLPSSW |
| DNRFELVMLATKRARQLATGGKEPKVA | EELADRLNTQMIHFVPRSKQ | DVREGIITYKIAAHAADLA | EIVAPEIASFQKSNDGTIK | ECWAVRKTATFMILGAVCTRACRF |
| DVALSDGVHKIEFEHGTTSGKRVVY | EEVLNNQELRLKNTL | DYASLRSLVASSGTLEFITE | EKRQIDSLTEEEEGWRRQLV | EEFNLKKGLVLAVMCG |
| EEKFFALIERQEGNT | EFELLPTDSGAVVIKSV | DYEASSSRCSSA | EQCQTRYLLTAPCDSPFVPTDLAAKLSQAMEEANARIAMP | EGEGIGSSRKGMADPIMAG |
| EFDKVLLVANGEDI | EFKNAMSVYINDIAEGLS | DYGCQVFSIIDFDDLVSFL | FDGDGDRLMMVD | EIEVIALTINHQLRGEDS |
| EGDNEASASAPPAAKRRKTDT | EFLRRAKEMQPDIPTKSSIMV | ECDSLLLAGLDKSAKH | FKSLDKAASKGLIHKNA | EIFFSDKARNGLFEG |
| EIAQLRRDYQDVTLKELGEMVSGG | EGRDLFNGNVDV | EDFGFSEIVVKLSTRPEKRVGSDDLWDRAESVMLEVLKTIEEQ | FPVKYFDKDAYLSQTGQLY | ELNLSGVVQSKAIEIINQAMDN |
| EIQKVTDDAVKEADRLASV | EGYELKQRLTQV | EEYQRRHNPIWPELEAV | FQHLRDEAGNSYSPEDYALHQAADG | EVIKHTTNNVTPPP |
| ELRRALGQDVTYSVA | EIYTGQLNGTEVALLKSGI | EKITQGTEGLNVPNEPIIPFIIGD | FQSLVRSPELAHMR | EWQCGTTQVDFNLPERFGAFYI |
| ELVERAAGSLPGWDCDIIEAHHVHKQDAPSGTALTLGEAATGS | ELAAAIDATFGSFEEFQ | EKKTSVLNSFQVFSYYM | FYRNDSSITNLTGN | EYYAKEVYKIDMPSFLPSE |
| EPTYRSNVNVK | ELAKQRFAAVGIDVEEALRQL | EKQHEFSHATGELT | GGIKEKGHPGGGDHDAWFV | FATLAGAYIHEA |
| ERSGEKLARRILSPAE | EPLCTPVVTCTPSCTTYTSSFVFTYPEADS | ELSLMDVKGKKTKK | GVEFKVYKNSLTRRA | FGDQVFTWQQYDEIVENE |
| ESMREEYRQEQQS | EPLGPNGRKMAE | ELSQLWFRRRYHIFFGYWL | HDTPTGSEWLE | FVVCYASESEEK |
| FHTMGQRKGLHIGGIKEKGH | EREERMKEGSIIMHPAPVNR | EMDFYSSECHSHTYEIVLNS | HITADGFYRGKKVIQTKT | GALHAAKRLLG |
| FPEKVKNITNKTGLDLQGVLGV | EVVHMCPTIGSNVEEIILP | ENPEFNDTDKLRKVINIM | HMTRLLRIKEAPFTLLSNV | GDYYLKPFVSAEPEVRVVE |
| FRLAAAGEQGFAFRVDYIEEL | FDRGGYLYHGRVKALAEAAREAGL | EPNKKKVGTITTAQLREIAQ | IEALADPRDVVIGLSTSGMSNN | GEKFEFDSVLMVVNGEDVKIGAPV |
| GETLSPLKIAGLVTLIGGILLV | FKIKYTEKGPQMDFESKNGQSLTKMACDNTQKSS | EQRDKRTRVGAP | IEIEVDGKKKRIGITRLHLEEDAGKSTHTADGSLVDYNRQGMP | GKYVVEGDLRREIGIAIKRLMD |
| GGEGRSPIGRAKPVTPWGK | FKPPKVNFTTRIYHPNI | ETAIVDTSKEAADLYLLQS | IEYLKNKILNLRIFEDEEG | GRSSRQAPDIDGQVYIT |
| GGGPVRVEGVGRDSVQGDV | FLTRKNGNKPEDERDHLTVVGA | FDEIFKRQIEALADPRDVVIGLSTSGMSNNVFHA | IGGDMVEYLPKWY | GSVSSTGSSSNTD |
| GGTVNVVETFMSKN | FNFILALVLFIGLAYYQGTPTST | FNIWGTVRTNRFDIIEESFKGLQPFWFRV | IGIQIEFAKVILAGEVTTPV | GTSADNRVSLTN |
| GHCQAPLNAFSFVLPGVNDRA | FPESQVCCGQPAFNSGYTKETIKAAKNMIKAFETAEY | FRSNQHIYAQVIDDVNGVTL | IPNIHISGEIKFE | HPEIGEDLEALLA |
| GKTAGRGTKGTKARYQVPER | FWMPYFRSLGVN | FSLSTLLPVFL | IPPSIGTPENVSTTIVDTSARPQDRTTLRKEIRKCNVILLVYADHYSYERVALFWMPYFRSL | IDHINGVLFYD |
| GREEQLRIENWMKA | GEIIGGSERDTDYDYLKQQIISQHLD | FVVLKKGLARL | IRLHGITDIHRRSFAPCVGQSV | IDTHIHFISPQQIDEA |
| GRKDLEIVILRG | GHFRFHWRPTGYIHRLPSLKKG | FVVVEDLGFEPDGEGEHILVRILKNGCNTRFVAD | ITEKGQRILAKRPFPPGQHGPSARRRQVSDYGLQLLE | IFMYPFFFNFFLVNAGAFL |
| GWTDVDLSDRGVEEAIWAGK | GIAIKRLMDLGCYRG | GALVLNVGTLS | IVRTKTGVVRPDG | IIHLELKGAGYFRAGDLAVD |
| HACMHLLYFRFFHKLLRDA | GIDTHIHFISPQQIDEALASGVTTMIGGGTGPAT | GDLSRNRSVGLQLAAGRSL | KATFVAEKNQQVAFR | IYVIWGPITERKKRRKGLVPGLVNLGNTCFMNSL |
| HGHHPHGHHPHGHHPPDN | GIEIDESLLTGPTAVAFIKGEA | GKKVAKVDASGLWKLYTRDGKQEPNATAISL | KGVVLFGDTRNQQDNGQIPDFPKD | KDRRVLQMLRENLEEEATVMKDVPG |
| ICIQLKALTLGA | GITMLTLTPDILFELKSRS | GLVDENVVQQEDIVEDEPLFAAFD | KLAAEGKGKRQ | KEETGFTEESNKNPIVFQP |
| IFLSVAFAATTYWAVGLDGGLTGLLFYCLIILASFWSGSS | GLLGGSTGPALCNRLKSALDGVAC | GNTGALMALSRFRL | KNLVDAEVMLGC | KIREEIARLQDQLKQAETREAERIGRIALKAGLG |
| IHFPTDYPFKPPKVNFTTRIYH | GQNPFNPAMIGYVVLLISFPVQMT | GQCKLAGPEDLSVNDFNCT | KQRTLSIIKPDAVEKNVIG | KTYELTEFIVDILHVTDVGASLKGNAT |
| IHRAICGSMERFLGILIEN | GSIASIVDVNLTVPEHHT | GQVLDAFVEVTLKDDE | KTALALNCKIHKYSRFDRKNYFYP | LAAEGMSPVLGYG |
| INGISLQVNMYTA | GSVLSWASSGSIGYKGTKKKTPYSAGIAAEA | GSGWEAIEASYRALLLNGARRAGVMDVPKAMPSAAAG | KVAKRYLQLLENLENNAKFK | LACPVCKGELKLNDAR |
| INLNFSIVCVF | GVAFGPRPKVYNLSM | GSRMGGVDKGLQTFRGAPM | KVVAEVVAQGRGDKVKIVKFRRRKHSR | LARSSPDRKIACRCLKSLATSIKSINMGKVSGVPGKCGVSVPFPISMSTN |
| IVSNKFINTMSQVPTITQLLPLPASEDDDLKRKAWDYLYE | HAASTGQSASETRENANSN | GVVQDIGSDPTLPRSDREC | LAKMQERGLPYISVL | LDRKTLADLAYN |
| IYQGEPNAFATGAFRNDSLVAVSTGLLDSMTEEEVAAVLGHEVAHVANGD | HIAREMEIEQAP | HFDYFSGQPITASNGSGFVIEQNGL | LKGFKRPKKIEFNTEAST | LEPGELPKSFEASGIS |
| KAEDPFQCLAACVILTQALK | HVPVYIQEDMVGHKLGEFAPTRTYKGHAADD | HLMKILTERGYSFTTTAE | LLGYACAMELDSNGRVLVPPTLRNYANLDK | LERQRVELQQEVEKLARENSSMRLELDALRSKYE |
| KDGDISYNPNVPSYSAKYQLNN | IARLQDQLKQAETREAERI | HNPAVASPHCQDYAYGYQQCIS | LLGYFGLDRMLP | LESVKAGQLIEVNLDLV |
| KDISLSDLSISIKIFSDCL | IATVTGRYFAM | HTTNLVPVALVGGPEGTALK | LLPWLRPDAKSQVTCRYEGGKV | LFIPGHHYQKDEVVQFSDAAG |
| KIAKRLKELSQKIDSNKEYALSDAID | IDIIADKKIVHKNKAAR | IMQPYDMPAGAGTFHPA | LLSALGGLMWYLQFFFYAWGHARI | LGMDATRVRLLVDPATRRNTHR |
| KKGIRCIVIPGTQN | IDPGSQRTGVGVID | IPYVRFAGELVVVCTAIVGA | LLSGLVYLVRIGALKWTPNH | LGWEVWLDGMEVTQFTYFQQVGGIAVDLVSAEITYGLERIAMYLQNVDNVYDIVWSEFNGEKIKYAD |
| KMAGRMGAERHTTLNLTV | IFSAAAAVFAGSAAAVGVSGSA | ISGHAFEIGEGG | LNIDWYRGLIQGKQMVM | LLVANGEDIKVGAPLVEGGK |
| KMKWGMEMRRCLLQSLQQIE | IKNIAEKIGLTEEDID | IVEISRIEAVVERSGEKLARRILSPAE | LNIILRSFLEFNHTYS | LMHGVLVWKVIVG |
| KSTNRPYTSRYIGSLVADFHRNLLKGGIYLYPSTASHPDGKL | ISAIILCVLILTLSFLLGE | IVPDHFTPNKDIKAAEQVK | LPAPKSFPFVI | LMVSLLTCIGQLCQKQA |
| KTAALARQHDDANVLCLSSRFVVPEENIKIVDEF | KAFFTTSQLSLQDKLQNAIALGTSPIVRGLVDFEGAMKTI | KAFGSSLALTGSPVGAMKVFL | LRSLETYERLEADKKKQVHKKRKCPG | LNYVKLDGQVGIIG |
| LALRHSAGSSMGM | KDKVVQVEDSKPV | KARRRIKRWRR | LVGPSGCGKSTLLRMVA | LSGDGAYDLAINLCKKPLTPRYAHLR |
| LGVYSFGLFTTTIFANAGQVVTGNPTPHFLSVCRPNY | KEANRMVEEVIIWDE | KDQFVNNKIQPVLPQITIPPSI | LVVSLSIFAVFGSQGVKQP | LSQYDLRIKAVDPSDLGID |
| LLDVGANVDCKPQNLQQFAI | KELVFQYIFIDQLELPPRIYNCLKKSN | KEGSNVTIVTFSIQVKLALD | MCHRQGGESETFAKRAIESL | NAGTAFRPLTAVLALSG |
| LLEHCKPDVIINTGSAGGLA | KKADENDIRLPGEV | KKERKPTVDAEEA | MEYLENPKKYIPGTKMIFAG | NALPKGTSDEAIEEAQS |
| LLIDDIQFFSGKSRTQEEFFYAFEAL | KMGIKAEEFNL | KNGQLAGHDSS | MEYTTTIPIGIKNTIN | NDAGAFIELQGTAEG |
| LQIKNLRISDTGTY | KPLVVRLDGNKVEEGRAILA | KQPNRHRGNRPPSVQGLKTQTTGH | MVSGGKISKSGINHRLRK | NEQNVELNRTSLYWGLLL |
| LREIEEGLINNQILDVRERQEQQE | KPYHRLVPSLLARIPVLIYA | KSNITQTRKASI | NFLTLFYTATVLLFTIPVLYE | NGLSLTSSMGFSVLDGVMMATRPGNLDPGVVLYLINNEKM |
| LSELDRDSSRLGLPRSVRES | LALLTSLAYFY | KTTILYRFLTNEV | NGVYVRMAVLKRALPNVLG | NNMLYPREDKENNRLLFECRT |
| LSMTPLPRAENCCGFGGTFSVKMTPIS | LARTMNGELSVF | LAMCSVEGGVEIEVTAEENPDALAKVPVDAVKGVDLALAREIAEKGKLPA | NIFKKEYAIVNVGTLEERF | NVELIPGRGGQ |
| LVQFPELIIMGLSEGVVPL | LKQAKAWLEDVVKLKRPIPI | LEGILILEDPRLCELMDIKLFVDTDADLRILRRMQRDIKE | NKSGYRRFGWRSVFQFCVPFSLGALV | NVQDPIVVGNTFGRVRAMTNDLGRRVKTAGPSTPVSI |
| LVSHGIALGCLV | LLGLAALGFISHNT | LFRKYDMDYCC | NQTLAVDGTALLKCKATGDP | PAGSSQKAREEKALLPLELQDD |
| MGFSSQKTYDI | LLTRSIQTAHIALME | LGISLHNFPEGIATFVTASSNLELGFG | NSDLNIERINE | PELYGMALRTVARTL |
| NAPLDRIIPVDVFVP | LNKSSYPSKRLIIVGSITG | LLAAQGSTRSAVGVIG | NVKTPLNREEVEKH | PIWVICYVPVIMSVLNILPA |
| NDKELMITAALPGSGEWGT | LPKRLPAHLLRVARLPRIEK | LLQVVGEGESAGAI | PLDAALHALQEEQARLKMRLWDLQQLRKELGDSPKDKVPF | PMTPPTCRFYPTCSHYGLEA |
| NGDYISDALAAQVGGIGIAP | LSMPGQHNALNATA | LNLRIFEDEEGKLNKSLKDVNGELLV | PNPEAPREGVVV | PTKPLPNDKLKKEIENFKF |
| NGQIPDFPKDKIKIYC | LSPAEWQHYQQHQQPIRFLAKRFAVKEAAAKAFGTGIRNG | LNRFFKTGRYEK | PRDYGKSSSGFDKTGPFRAQFL | PVSGTAILEEIRRA |
| NGRKTNIPSYTVRVGE | LWDRAESVMLEVLK | LPLKEKFNKSSDLELKGS | QAANGLFAVLRVTAPLAQSDKKLLSAFADE | QAEGFSYTDANKNKGITWGEDTLMEYLENP |
| NGWIEIEVDGKKKRI | MASTMQRTSSSAASNERQLS | LREIAQLRRDYQDVTLKEL | QALRKLTATLARTNT | QEEQGMSAEGVEGYG |
| NLAAQQDSTNVRLYSLLLASKG | MDIAVRAHLNGWKFIF | LRTRVEVVGELISKPYIEI | QHRVSEGQTLRLEKLDVETGAT | QELLIPFLKMLRSIGGGFL |
| NLLPPPEPKESW | NAALSLTMSPSVKLETQ | LSIHFPTDYPFKPPK | QQHPDKEIVSLNPFMCPCL | QGVIGNQQVNT |
| NVHCITVNGVFDDCQDLV | NDSSITNLTGNLK | LVIPMLTEERRR | QRAAELAAELGV | QLLNIDWYRGLIQGKQM |
| PAIVKPKGWGIFSGILLDQ | NRNLGELGETSGGSILVRE | LVPEDELPLCESLKDTEARVVPYWADMIVPAIKEGKKVLI | REKGVGSLQTASERKEMALECQQLCAKYQVP | QVGGKDPLVPEENDKTTVIA |
| PCGCGHRGCIENYISGRGFE | PATFLRSLGKKPWAAAYVAPSRRPTDGRYG | MLCFLEYNLIWMISSYI | RELLDAMKRKEKLVAMGHL | QVKEETEAWERMVDEYRD |
| PEGEVFNPEFHQAM | PEITIKSQSVRLRFIKILTGNIRNVL | MVGHKLGEFAPTRTYKGHAADDKKT | RFGDTHVLCTASVDEKVPPHV | RADFFVTDDNQIYINETNAM |
| PIAPLFDYKEGHLKPLC | PIGSGLGVRNPYTTRIIVE | NTFFPWIEKQGLTVGIIIL | RFLGVYSFGLFTTTI | RADRQPEFTQIDIETSFLND |
| PISNHPAPEYWCSIA | PKPACTLEHRPL | NVVMNDAGAFIELQGTAEG | RHYGALQGLNKAEMTERYGE | RDMRNSVNRLVNCET |
| PLELPMVTTIQMSSQLSGR | PLDEVPDGKDEHDNVERHVFGARR | PGRDPRATIQEQGTLQI | RKLVIEQGINAREIEVAVL | RFVNQSLGTCIEAYGNG |
| PLKLVDVADLMQEVEFKVF | PLLQDSHIGELVETQKQ | PLLGLKGAAFVGHGSSNANAIKNAIRVAAEYVEHRVNEAIAR | RPGPGAAPAAGG | RFVPFCQKSKVT |
| PMACGDGGYPSVLDSQSC | PNKQVVELNRTSLYWGLLLIFVLAVL | PNPNKQVVELNRTSLYWGL | RRAPSLMIKPTSF | RGAVEDLELEDVLLQGFADI |
| PTPTESLRRLHPHLGRTLI | PPTLRNYANLDKKLMLVGMGKKFELWSEESWWASVADLDVDEE | PRDYGKSSSGFDKTGPFR | RRVTQVVRDFLHAQKVQPPVELFVDWLAVGH | RKAYLESFRKGFKQQIENT |
| QHAQQQAGSADASANNAKDDDVVDAEFEEVK | PQLRHAGAIFMGRFTSESLGDYCAGPNHVLPTSRTARFASPLGVYDFQKRSSLIRVSQAGAQKLGRI | QALFNAFMALLDPGDEVIVFSPV | SAAKTHYMSGGQVK | RLKYGLKRGPSTEGA |
| QKHLNLREDIPAFDINAACAGFVYALSIADQYIRNGGAKHILVIGVDSLTKVVDWKDRSTCI | PTRGEVTLLDRELSTISEA | QAYLAGRIQKKL | SAEPWLQLISAVIIIGTAFWMF | RLVIPMLTEER |
| QKVINVKEVRLSPTIEEHDFNTKLRNARKF | PYFAANELGGAPAFD | QSASGDPEVDKKI | SGCVPQAQPRQEYMKGL | RMLAAATAFTRICAGQGEG |
| QRVQIFPIDSAIDVVSP | QCFLETDLFNQG | QSLVLVKKLLAIAISSITY | SGIQNLMEYVLEF | RTPALRARLFTEDER |
| QSQLPAADVADTVI | QDIGSDPTLPRSDRECPKC | QVWRVSQFLGFHKDTGDRPIS | SILLLNQVTEII | SAGPSKPQEDPES |
| RAIKDTAASPQLAHAAQQLFRL | QPVTLFLGVGVLGGYTTFST | RAIVSGGGKKPYRQ | SRFDPDRWLPDRTSAVARQAFTAFG | SAIKRVKVNEKANIANSQA |
| RAKRALLKQRQATQRVS | QVASVSVGVYRGEPVLDL | REDIPAFDINAACAGFVYALSIAD | SSVSALLLKQQGYDVVGVFMKNWD | SGKRVVYVDGK |
| RENANSNAKLGI | RIKYEPEEKDQSKIIHLELKGAGYFRAGDLAVDSSIEIMNPDLHIATLNEDANLVMDLEIQR | RLLVHMGLLKSEDKVKAIANLY | STDVCQRWWKYMTDVMP | SHFKILVKSVFENINKLDE |
| RGDLPGAEASA | RKQQGHRQWFTEVKIT | RSQAQQPQKEAALS | SVAKRAVEKGVKEVVFD | SILTAVGNDYGFDE |
| RGFSFTHNGPL | RPSGFDREKNNTPIQWKLGSD | SDLWFCPESKIWTVKSSSIHRGLVVTTGGTFRSL | SVLIFANKQDVKDSMRMVEI | SKGIKNLGKHIENVNKFGV |
| RGQRRSHDALSAKQLSTDP | RQSNRVEVKNMNSFRFVEK | SLGNVILPADVVKNLGADVLRLWA | SYVRSPYDPDMDNRYLTTYNQG | SKLRTRQMRCPDYRLRLARSTLTLVPLLG |
| RHLTEFWMIDAEM | RYRATAGFRNI | SLHRDLDATRESGLS | TEDDLKDLENEI | SNANRGRVTVEYVMLDHINDST |
| RLILERLQVVYTVGYSL | SAPAGAAPAAPRAPLAPTG | SLQIVDYFIRIVYTVRYAPN | TGLSYKIDAAG | SQLEFPLSQDPQPS |
| RLNVFRSNQHIYAQVID | SEKKRISLADQIKQRARAW | SNKYRFFSYGDAMLITPES | TGPCESNGDKGLGGIKGGFVHQRMASKIGRWYSR | SSLFGISFSDHLKSEFSSS |
| RPLDNLLMEARTLVATGTR | SEVTANSMRPLAAAARSAAA | SRTARFASPLGVYDFQKRSSLIRV | TIVRFMLSRDKVISEEELKEIQDFAQFSYGKNLALYHLSFKARTEKEVREYLKKYDIDKNIVSQVIA | STGVDDVYQHGR |
| RRAPSLMIKPTS | SHNLNIHTCSDLWFCPESKIWTVKSSS | SSGVGWLITFCSDTGDC | TKVVDWKDRSTCILFGDGA | TAGFPIILIPL |
| RSWFPSAPGSASSSKVSGV | SLLDRVQVVYY | STGQSASETRENANSNAKLGISLEESLQILNVKTPLNREEVEKHYEHLFNINDKSKGGTLYL | TLIQGVVNTFVVFLARVVGYFVDR | TALLSAIKLGAKIIH |
| SILLLNQVTEI | SREGLLSMLLSVLQAPIRNL | STRPRRKERKNISSGVAHVLAS | TLLKLAAKEAGIEIDESLL | TGPNAIAFSNEDVVAPAKVLND |
| SKECCSGVQGLNGLARSSP | SSTLRITDCRLKG | SYFQGNLEVPVLHGIDLQVN | TYHTEGAGGGHAPD | TGVSAEMFAMPRDLKGSKKDGIPEDLDGNLE |
| SKIWTVKSSSI | SYGVLSGPSFAREV | SYISDVVQPVGWL | VAYLTYAERRVIGLMQLRR | TISEAERGSLRNHALGFVYQF |
| SPNGEMILYAMNQGGQGKLA | TASWVLFELFEYHLLAFLCHF | TAEIIAQACGCDITFDSRLRELDMGV | VESEPNGTQRTFQTQENATP | TLAVVRHWDNI |
| SQYFYPIATEGFLEI | TEIINDWQEKDDSQ | TERPPVVTIMGHVDHGKTTLLDTLRNSRVA | VFPIAKRESLGMADIHK | TLRLHQRAAELAAELGVKSLHV |
| SSAHEISTIKT | TELICYQDAMAFPIREGIP | TGGSVFYESPFT | VGIIGNGAGLVMST | TLSSTKPLQLDS |
| SSSSFKSKIYRLF | TELRKVGATVE | TGLPIIETKANDISAYIPT | VIAKALEKKSELKRCHWSDMFTGRLRTEI | TMQANPNSLGI |
| TADAYVCACDIPGIQRVLP | TSNDACLSIVHSLMCHRQG | TGSSSNTDSSTKNAG | VLSTLWLGALAALLIEINR | TVKIGVWLRQKRGSGKIAFLQLRD |
| TDDPHMCKRFEDLGCVAVMPLAAPI | TVEKGGKHKTGPNLHGLF | TGTLNLSGIIEAQKTLPWW | VNIASYQVSPNDVVSIREKAKKQS | VDVRLSELGEKEAKTAGQL |
| TEELAQRVEQAF | VATTDKQKGVLFELATAKK | TPNYGKFVAEPFERG | VRSDSAASDQTSKSYDRGYSDKNIVAHSSGSRGS | VGTLEERFEDGA |
| TKQQVVTEIADKLRASK | VIIILIFTLLNIPF | TPYFTTKADGTGLGLAVVQNIIEQHGGAIKV | VSAEMFAMPRDL | VISVPVEVCQKIRQTKENGGR |
| TRQTFLRVQERQGQSRRRKGPHC | VKLRAILKFLEEGNKVKITLRF | TRHTEALGELTRYLGIGDY | VTEVFAVVCFREKFQ | VLAKMYACKPFYVFYYNPENNTLLSVEIQGVGEDSEWFSN |
| TRSVAEGEINSVLDIAYPHEDDTVTKPTDI | VNKITYGACPKYVKQNTLKLAI | TSAYVPCHPELLRQ | VTEVTIRNRYKELT | VPAIKEGKKVLISAHGNSM |
| TSLYWGLLLIFVL | VPAALSITIASEKLHFFSS | TVDQAIERAGTKAGNKG | VTPQAVLAASKPEF | VRRGFEGGQMPLYRRLPKFGFTSRKAA |
| TSPEKNERVRQMRPVCDCQAHLLW | VSAEPPAEFWPALESFLDDM | VEGGLAKMRDSISNT | VVSDEARYHDADVTAFGYEY | VRYAPNILLHYQLFCITKKF |
| TVVSFTSDHASDGRNTAANDKA | VSASLSYLLYSDILL | VGAPLVEGGKVVAEV | VVVAGGDGKAAAARRRRRLELRR | VVISCTSSPELMLDFEQAQ |
| VLNSFSSIWLLI | VTLDCDVIQADGGT | VMLAVEARTLSTDER | VYDQMAEPRWVISMG | VYVRMAVLKRALPNVLGGMKHELF |
| VNRGDVCVTQDHALA | VVYRLGFATTRAQARQL | VSGACCRFSPPLRR | YAEDGEAETDMNVVMNDAGAFIELQGT | YFVGAGPGDPGLLT |
| VVHMCPTIGSNVEEIILPKTHFFMWDIVRPEALS | YDTMLQLTKKY | VTGMGDNPSLSELIG | YELFPSSRELNMTWYSVVKL | YGVDEAGRGPLAGPVYAACVVLDPADVIEG |
| WLLTYGLGLLV | YFFISKEKFEEMIEKGEFLEYAQIY | VVRLEKLEIATGEKFEFDS | YETENLSDNDIIGTSIYNV | YKEEEMPAMLAWRHTSQKAR |
| YFLDEYKKGRTPNPD | YGEKATLRHWLGVAAIMFG | YLSAAPVLSTLWLGALA | YFPPGSTLTDK | YNQYIRIRHILNPS |
| YLRGLFSEKAYGRKYVG | YINETNAMPGFTAYS | YVIKPLAQGSS | YFQGIRVYLKEKKYSDCAW | YNVTPNKAGKYFVKIHCFCFEEQLLKAGEKVLMPVTFYIDKDFENDPEMRDVKVLTLSYSFF |
| YNHKEVFNAIVEKT | YKQVEKPVYDYTLHTR | YVRMAGDGLEEPPVEPGMNVK | YIFRYYILYVRQPSIKKLV | YVPRAVLVDLEPGTMDSVRSGPFGQIFRPDNFVFGQSGAGNN |
| YNLVGIKPGKESYMRLNEKALD | YLKIEFLSTPFVILFFVLEQFA | YYVSHNRFYEQVLVPKDPAF | YLKLDFKLKNQVVPFESYQKQVSQIHNAVKSKSVEEKDWLGW | YVRTAVDQHLGPGAMVMPQA |
