# Supplementary material for: AntiAngioPred: A Server for Prediction of Anti-Angiogenic Peptides
Source: PLoS One. 2015 Sep 3;10(9):e0136990. doi: 10.1371/journal.pone.0136990 (PMC4559406; doi:10.1371/journal.pone.0136990)
Supplement: S5 Table — (DOCX) [file pone.0136990.s007.docx]

**S5 Table**: All (257) anti-angiogenic peptides extracted from literature (literature reference is given in header line of the fasta formatted peptide sequences)

>Endostatin: An Endogenous Inhibitor of Angiogenesis and Tumor Growth (http://www.ncbi.nlm.nih.gov/pubmed/9008168)

MARRASVGTD

>Anti-Angiogenic Peptide US 20110144022 A1

HNRTPENFPCKNL

>Anti-Angiogenic Peptide US 20110144022 A1

DSSPVSTEQLAPTA

>Anti-Angiogenic Peptide US 20110144022 A1

SSTSPHRPRFS

>PIGF Exon 6 Anti-angiogenic peptides US 20030186868 A1

RRPKGRGKRRREKQRP

>P6V8 Anti-angiogenic peptides US 20030186868 A1

RRPKGRGKRRREKQRPCDKPRR

>Anti-angiogenic peptides US 20030186868 A1

RRPKGRGKRRREKQRPSDKPRR

>Anti-angiogenic peptides US 20030186868 A1

RRPKGRGKRRREKQRPDAVPRR

>P6P7C Anti-angiogenic peptides US 20030186868 A1

RRPKGRGKRRREKQRPTDCHLCGDAVPRR

>P6P7C Anti-angiogenic peptides US 20030186868 A1

RRPKGRGKRRREKQRPTDCHLCGDAVPRR

>P6V8 Anti-angiogenic peptides US 20030186868 A1

KGRGKRRRCKQRPSDCPRR

>P6V8 Anti-angiogenic peptides US 20030186868 A1

KGRGKRRRECQRPSCKPRR

>Anti-angiogenic peptides US 20030186868 A1

SKRKSRPVSVKTFEDIPLEEP

>P6V8-3NCS1-7CC Anti-angiogenic peptides US 20030186868 A1

CKGRGKRCREKQRPSDKPRR

>P6V8-3N Anti-angiogenic peptides US 20030186868 A1

KGRGKRRREKQRPCDKPRR

>P6V8-8N Anti-angiogenic peptides US 20030186868 A1

RRREKQRPCDKPRR

>P6V8-3N and P6V8-4N Anti-angiogenic peptides US 20030186868 A1

GRGKRRREKQRPCDKPRR

>P6V8-7N Anti-angiogenic peptides US 20030186868 A1

KRRREKQRPCDKPRR

>P6V8-3NCS-1C Anti-angiogenic peptides US 20030186868 A1

KGRGKRRREKQRPSDKPR

>P6V8-3NCS-2C Anti-angiogenic peptides US 20030186868 A1

KGRGKRRREKQRPSDKP

> P6V8-9N Anti-angiogenic peptides US 20030186868 A1

RREKQRPCDKPRR

>P6V8-3NCS Anti-angiogenic peptides US 20030186868 A1

KGRGKRRREKQRPSDKPRR

>P6V8-3NCSC13C18 Anti-angiogenic peptides US 20030186868 A1

KGRGKRRRECQRPSCKPRR

>Anti-angiogenic peptides US 20030186868 A1

RRPAAAGKRRREKQRPSDKPRR

>Anti-angiogenic peptides US 20030186868 A1

RRPKGRAMRREKQRPSDKPRR

>Anti-angiogenic peptides US 20030186868 A1

RRPKGRGKRAAAKQRPSDKPRR

>TIMP-3 Loop-5 Anti-angiogenic peptides US 8431396 B2

KIKSCYYLPCFVTS

>TIMP-3 Loop 6 Anti-angiogenic peptides US 8431396 B2

KNECLWTDMLSNFGYPGYQSKHYACIRQKG

>TIMP-3 Tail Anti-angiogenic peptides US 8431396 B2

GYCSWYRGWAPPDKSIINATDP

>TIMP-3 N Peptide Anti-angiogenic peptides US 8431396 B2

RGFTKMPHVQIHTEASESL

>Novel antiangiogenic peptides US 20020086420 A1

CKITRCPMIPCYISSPDECLWMDWVTEKNINGHQAKFFACIKRSDGSCAWYRGAAPPKQEFLDIEDP

>aa 129-138; partial loop-4+loop-5 Novel antiangiogenic peptides US 20020086420 A1

KITRCPMIPC

>aa 129-145; partial loop-4+loop-5 Novel antiangiogenic peptides US 20020086420 A1

KITRCPMIPCYISSPDE

>aa 128-167; partial loop-4+loop-5+loop-6 Novel antiangiogenic peptides US 20020086420 A1

CKITRCPMIPCYISSPDECLWMDWVTEKNINGHQAKFFAC

>aa 146-167; loop-5 Novel antiangiogenic peptides US 20020086420 A1

CLWMDWVTEKNINGHQAKFFAC

>AA 128-175; loop-4+loop-5+loop-6 Novel antiangiogenic peptides US 20020086420 A1

CKITRCPMIPCYISSPDECLWMDWVTEKNINGHQAKFFACIKRSDGSC

>aa 176-194 Novel antiangiogenic peptides US 20020086420 A1

AWYRGAAPPKQEFLDIEDP

>Loop 6 Anti-angiogenic peptides US 8431396 B2

ECLWMDWVTEKNINGHQAKFFACI

>Loop 5 Anti-angiogenic peptides US 8431396 B2

TRCPMIPCYI

>Antiangiogenic peptides polynucleotides encoding same and methods for inhibiting angiogenesis US 6057122 A

INGSLDKRLLPDVET

>Antiangiogenic peptides polynucleotides encoding same and methods for inhibiting angiogenesis US 6057122 A

INGSLDKRVQDCYHG

>Antiangiogenic peptides polynucleotides encoding same and methods for inhibiting angiogenesis US 6057122 A

INLEACLGRTLMD

>Antiangiogenic peptides polynucleotides encoding same and methods for inhibiting angiogenesis US 6057122 A

INLEACLKRGRT

>Antiangiogenic peptides polynucleotides encoding same and methods for inhibiting angiogenesis US 6057122 A

MFSPILSLEIILALATLQSVFAQPVICTTVGSAAEGS

>Antiangiogenic peptides polynucleotides encoding same and methods for inhibiting angiogenesis US 6057122 A

INEFLERSGIPRQRNQ

>Antiangiogenic peptides polynucleotides encoding same and methods for inhibiting angiogenesis US 6057122 A

RCRLAERRQIAK

>synVB1 Antiangiogenic peptides polynucleotides encoding same and methods for inhibiting angiogenesis US 6057122 A

SVSRAGSPSGGPFC

>Antiangiogenic peptides polynucleotides encoding same and methods for inhibiting angiogenesis US 6057122 A

ALARQPLTGSPPNERAFFCSSLRR

>synVB2 Antiangiogenic peptides polynucleotides encoding same and methods for inhibiting angiogenesis US 6057122 A

SVSGGGHHHHHHGGG

>Antiangiogenic peptides polynucleotides encoding same and methods for inhibiting angiogenesis US 6057122 A

LVPRGSRAGSPSGGPFCALARQPLTGARLMSGLFFALHET

>synVB3 Antiangiogenic peptides polynucleotides encoding same and methods for inhibiting angiogenesis US 6057122 A

SVSGGGHHHHHHGGG

>Antiangiogenic peptides polynucleotides encoding same and methods for inhibiting angiogenesis US 6057122 A

RAGSPSGGPFCALARQPLTGSPPNERAFFCSSRDV

>synVB4 Antiangiogenic peptides polynucleotides encoding same and methods for inhibiting angiogenesis US 6057122 A

SVSGGGHHHHHHGGG

>Antiangiogenic peptides polynucleotides encoding same and methods for inhibiting angiogenesis US 6057122 A

DDDDKRAGSPSGGPFCALARQPLTGSPPNERAFFCSSRDV

>Angiostatin protein US 7365159 B2

YPYDVPDYASL

>Angiostatin protein US 7365159 B2

IARALFEKKV

>http://molpharm.aspetjournals.org/content/55/2/332.full.pdf

SPWSSASVTAGDGVITRI

>http://molpharm.aspetjournals.org/content/55/2/332.full.pdf

SPWDIASVTAGGVQKRS

>http://molpharm.aspetjournals.org/content/55/2/332.full.pdf

SPWSSASVTAGDGVDITRIR

>http://molpharm.aspetjournals.org/content/55/2/332.full.pdf

GDGVDITRIR

>http://molpharm.aspetjournals.org/content/55/2/332.full.pdf

GDVIDTDRDIDR

>Human Endostatin protein sequence Methods of producing anti-angiogenic proteins US 6797488 B1

IYSFDGRDIMTDPSWPQKVIWHGSSPHGVRLVDNYCEAWRTA

>Methods of producing anti-angiogenic proteins US 6797488 B1

DTAVTGLASPLSTGKILDQKAYSCANRLIVLCIENSFMTDARK

>Compounds for targeting endothelial cells, compositions containing the same and methods for their use US 7884183 B2

DPPEGLUGTKPPROH

>Compounds for targeting endothelial cells, compositions containing the same and methods for their use US 7884183 B2

TKPPRKRPPKTKKRPPKTTKPPRGZOG

>Compounds for targeting endothelial cells, compositions containing the same and methods for their use US 7884183 B2

TKPPRKRPPKTGZOG

>Albumin-Fused Anti-Angiogenesis Peptides US 20120263747 A1

MLQNSAVLLLLVISASA

>Albumin-Fused Anti-Angiogenesis Peptides US 20120263747 A1

MPTWAWWLFLVLLLALWAPARG

>VC053 Albumin-Fused Anti-Angiogenesis Peptides US 20120263747 A1

DLWIRETLTSPKSLTG

>VC054 Albumin-Fused Anti-Angiogenesis Peptides US 20120263747 A1

PTGERLRTCERLSYP

>VC055 Albumin-Fused Anti-Angiogenesis Peptides US 20120263747 A1

DLWIRETLTSPKSLID

>VC056 Albumin-Fused Anti-Angiogenesis Peptides US 20120263747 A1

PIDERLRTCERLSYP

>VC057 Albumin-Fused Anti-Angiogenesis Peptides US 20120263747 A1

SRTVRKTSRLWSSLSLNTCNNVHSKS

>VC058 Albumin-Fused Anti-Angiogenesis Peptides US 20120263747 A1

VIFEWTLLQVLSESDQDQSLEVFLT

>CF96 Albumin-Fused Anti-Angiogenesis Peptides US 20120263747 A1

RIFGESVSLRVQDWEW

>CF97 Albumin-Fused Anti-Angiogenesis Peptides US 20120263747 A1

GHRATSDLASTGEESQD

>Albumin-Fused Anti-Angiogenesis Peptides US 20120263747 A1

VGSGGCMFGNGK

>Albumin-Fused Anti-Angiogenesis Peptides US 20120263747 A1

HKLINTEGHHS

>Albumin-Fused Anti-Angiogenesis Peptides US 20120263747 A1

VRSCMFGNGK

>Albumin-Fused Anti-Angiogenesis Peptides US 20120263747 A1

HGSTTLRDITV

>http://www.ncbi.nlm.nih.gov/pubmed/18307172

NGRKACLNPASPIVKKIIEKMLNS

>http://www.ncbi.nlm.nih.gov/pubmed/18307172

NGKKACLNPASPMVQKIIEKIL

>http://www.ncbi.nlm.nih.gov/pubmed/18307172

NGKEICLDPEAPFLKKVIQKILD

>http://www.ncbi.nlm.nih.gov/pubmed/18307172

NGKQVCLDPEAPFLKKVIQKILDS

>http://www.ncbi.nlm.nih.gov/pubmed/18307172

DGRKICLDPDAPRIKKIVQKKL

>http://www.ncbi.nlm.nih.gov/pubmed/18307172

DGRELCLDPKENWVQRVVEKFLK

>http://www.ncbi.nlm.nih.gov/pubmed/18307172

DGRELCLDPKENWVQRVVEKFLK

>oligonucleotides:DNA complexes containing DNA encoding anti-angiogenic peptides and their use in gene therapy US 20020151516 A1

LSSTCILVLVKSTY

>oligonucleotides,anti-angiogenic fragment from laminin:DNA complexes containing DNA encoding anti-angiogenic peptides and their use in gene therapy US 20020151516 A1

DRSTREPIYMSTI

>Laminin peptide concatame Mector:DNA complexes containing DNA encoding anti-angiogenic peptides and their use in gene therapy US 20020151516 A1

LSSTCILVLVKDILVLVVKEILVLVVKDKPI

>Anti-angiogenic peptides for cancer therapeutics (http://www.ncbi.nlm.nih.gov/pubmed/21470139)

FLSSRLQDLYSIVRRADRAA

>minimal epitope Anti-angiogenic peptides for cancer therapeutics (http://www.ncbi.nlm.nih.gov/pubmed/21470139)

IVRRADRAAVP

>pro-angiogenic A13 Anti-angiogenic peptides for cancer therapeutics (http://www.ncbi.nlm.nih.gov/pubmed/21470139)

RQVFQVAYIIIKA

>pro-angiogenic C16 Anti-angiogenic peptides for cancer therapeutics (http://www.ncbi.nlm.nih.gov/pubmed/21470139)

KAFDITYVRLKF

> Anti-angiogenic peptides for cancer therapeutics (http://www.ncbi.nlm.nih.gov/pubmed/21470139)

DFKLFAVYIKYR

>inhibited angiogenesis C16S Anti-angiogenic peptides for cancer therapeutics (http://www.ncbi.nlm.nih.gov/pubmed/21470139)

DFKLFAVTIKYR

>fusion protein carrying the amino acid Endostatin: An Endogenous Inhibitor of Angiogenesis and Tumor Growth (http://www.ncbi.nlm.nih.gov/pubmed/9008168)

MARRASVGTD

>hsurvivin:Dendritic Cell Targeting of Survivin Protein in a Xenogeneic Form Elicits Strong CD4+ T Cell Immunity to Mouse Survivin1 (http://www.jimmunol.org/content/177/12/8410.full)

FLKDHRISTFKNWPF

>msurvivin:Dendritic Cell Targeting of Survivin Protein in a Xenogeneic Form Elicits Strong CD4+ T Cell Immunity to Mouse Survivin1 (http://www.jimmunol.org/content/177/12/8410.full)

YLKNYRIATFKNWPF

>SPARC peptides FSEN:Anti-angiogenic SPARC peptides inhibit progression of neuroblastoma tumors (http://www.molecular-cancer.com/content/9/1/138)

CQNHHAKHGKVC

>Anti-angiogenic SPARC peptides inhibit progression of neuroblastoma tumors (http://www.molecular-cancer.com/content/9/1/138)

CELDENNTPMC

>Control scrambled peptides scFSEN:Anti-angiogenic SPARC peptides inhibit progression of neuroblastoma tumors (http://www.molecular-cancer.com/content/9/1/138)

KCGHKHQCAVHN

>Anti-angiogenic SPARC peptides inhibit progression of neuroblastoma tumors (http://www.molecular-cancer.com/content/9/1/138)

MEPECNLNCTD

>Inhibition of Angiogenesis by Thrombospondin-1 Is Mediated by 2 Independent Regions Within the Type 1 Repeats (http://www.ncbi.nlm.nih.gov/pubmed/10500044)

VTCGDGVITR

>Inhibition of Angiogenesis by Thrombospondin-1 Is Mediated by 2 Independent Regions Within the Type 1 Repeats (http://www.ncbi.nlm.nih.gov/pubmed/10500044)

VTCGGGVQKRSRL

>Inhibition of Angiogenesis by Thrombospondin-1 Is Mediated by 2 Independent Regions Within the Type 1 Repeats (http://www.ncbi.nlm.nih.gov/pubmed/10500044)

GGWSHWSPWSS

>Two Functional Epitopes of Pigment Epithelial?Derived Factor Block Angiogenesis and Induce Differentiation in Prostate Cancer (http://www.ncbi.nlm.nih.gov/pubmed/15958558)

TGALVQQQDP

>Two Functional Epitopes of Pigment Epithelial?Derived Factor Block Angiogenesis and Induce Differentiation in Prostate Cancer (http://www.ncbi.nlm.nih.gov/pubmed/15958558)

TGASSEEEDPF

>Thrombospondin-1 as an endogenous inhibitor of angiogenesis and tumor growth (http://www.ncbi.nlm.nih.gov/pubmed/12003665)

SPWSSCSVTCGDGVITRIR

>Thrombospondin-1 as an endogenous inhibitor of angiogenesis and tumor growth (http://www.ncbi.nlm.nih.gov/pubmed/12003665)

KRFKQDGGWSHWSPWSSC

>CD36 and HIV gp 120 Thrombospondin-1 as an endogenous inhibitor of angiogenesis and tumor growth (http://www.ncbi.nlm.nih.gov/pubmed/12003665)

KRFKQDGGWSHWSPWSSCSVTCGDGVITRIRLCNSPSPQMNGKPCEGEARETKACKKDACPI

>KV11 Inhibition of Pathologic Retinal Neovascularization by a Small Peptide Derived from Human Apolipoprotein(a) (http://www.ncbi.nlm.nih.gov/pubmed/19515999)

YTMNPRKLFDY

>TL1AL72-L251(rev) Two Functionally Distinct Isoforms of TL1A (TNFSF15) Generated by Differential Ectodomain Shedding (http://www.ncbi.nlm.nih.gov/pmc/articles/PMC2954241/)

YRIPIVRRLQRR

>TL1AV84-L251(fw) Two Functionally Distinct Isoforms of TL1A (TNFSF15) Generated by Differential Ectodomain Shedding (http://www.ncbi.nlm.nih.gov/pmc/articles/PMC2954241/)

YTYGLCTSSR

>Tumor progression: the effects of thrombospondin-1 and -2 (http://www.ncbi.nlm.nih.gov/pubmed/11520937)

DGGWSHWSPWSSC

>KV11 Inhibition of Pathologic Retinal Neovascularization by a Small Peptide Derived from Human Apolipoprotein(a) (http://www.ncbi.nlm.nih.gov/pubmed/19515999)

YTMNPRKLFDY

>Two RGD-independent avb3 Integrin Binding Sites on Tumstatin Regulate Distinct Anti-tumor Properties (http://www.ncbi.nlm.nih.gov/pubmed/10837460)

PGLKGKRGDSGSPATWTTRG

>cyclic decapeptide How molecular imaging is speeding up antiangiogenic drug development (http://www.ncbi.nlm.nih.gov/pubmed/17121909)

CTTHWGFTLC

>E-selectin-binding peptide How molecular imaging is speeding up antiangiogenic drug development (http://mct.aacrjournals.org/content/5/11/2624.full)

CDSDSDITWDQLWDLMK

>ADAMTS1 mediates the release of antiangiogenic polypeptides from TSP1 and 2 (http://www.ncbi.nlm.nih.gov/pubmed/17082774)

TEENRELVSELKRP

>LRR5 Decorin derived antiangiogenic peptide LRR5 inhibitsendothelial cell migration by interfering with VEGF-stimulated NO release (http://www.ncbi.nlm.nih.gov/pubmed/18373940)

QMIVIELGTNPLKSSGIENGAFQGMK

>LRR5C Decorin derived antiangiogenic peptide LRR5 inhibitsendothelial cell migration by interfering with VEGF-stimulated NO release (http://www.ncbi.nlm.nih.gov/pubmed/18373940)

SSGIENGAFQGMK

>LRR5M Decorin derived antiangiogenic peptide LRR5 inhibitsendothelial cell migration by interfering with VEGF-stimulated NO release (http://www.ncbi.nlm.nih.gov/pubmed/18373940)

LGTNPLKSSGIE

>LRR5N Decorin derived antiangiogenic peptide LRR5 inhibitsendothelial cell migration by interfering with VEGF-stimulated NO release (http://www.ncbi.nlm.nih.gov/pubmed/18373940)

QMIVIELGTNPLK

>Polyclonal Rabbit anti mouse K1 Anti-angiogenic role of angiostatin during corneal wound healing (http://www.ncbi.nlm.nih.gov/pubmed/15106938)

SPSTHPNEGLEENYCRNPDN

>Polyclonal Rabbit anti mouse K2 Anti-angiogenic role of angiostatin during corneal wound healing (http://www.ncbi.nlm.nih.gov/pubmed/15106938)

EKYEGKISKTMSGLDCQAWDS

>Polyclonal Rabbit anti mouse K3 Anti-angiogenic role of angiostatin during corneal wound healing (http://www.ncbi.nlm.nih.gov/pubmed/15106938)

EIPSCESSASPDQSDSSVPPEE

>Polyclonal Rabbit anti mouse K4 Anti-angiogenic role of angiostatin during corneal wound healing (http://www.ncbi.nlm.nih.gov/pubmed/15106938)

TTITGKKCQSWAAMFPHRHSKT

>Polyclonal Rabbit anti mouse K5 Anti-angiogenic role of angiostatin during corneal wound healing (http://www.ncbi.nlm.nih.gov/pubmed/15106938)

QEPHRHSIFTPQTNPRADLEKN

>Polyclonal Rabbit anti mouse B chain Anti-angiogenic role of angiostatin during corneal wound healing (http://www.ncbi.nlm.nih.gov/pubmed/15106938)

EKSSRPEFYKVILGAHEEYIRG

>Peptides Derived from the Histidine-Proline Domain of the Histidine-Proline-Rich Glycoprotein Bind to Tropomyosin and Have Antiangiogenic and Antitumor Activities (http://www.ncbi.nlm.nih.gov/pubmed/15313924)

GFHDHGPCDPPSHK

>(http://www.ncbi.nlm.nih.gov/pmc/articles/PMC2613512/)

TMPFLFCNVNDCNFASRNDYSYWL

>V14 Elastin-derived peptides enhance angiogenesis by promoting endothelial cell migration and tubulogenesis through upregulation of MT1-MMP (http://www.ncbi.nlm.nih.gov/pubmed/15632106)

VVGSPSAQDEASPL

>Tumstatin Peptide (http://www.ncbi.nlm.nih.gov/pmc/articles/PMC3114256/table/T2/)

TLPFAYCNIHQVCHYAQRNDRSYWL

>Pentastatin-1 (http://www.ncbi.nlm.nih.gov/pmc/articles/PMC3114256/table/T2/)

LRRFSTMPFMFCNINNVCNF

>Endostatin peptide (http://www.ncbi.nlm.nih.gov/pmc/articles/PMC3114256/table/T2/)

HTHQDFQPVLHLVALNTPLSGGMRGIR

>Endostatin fragment IV,IVox (http://www.ncbi.nlm.nih.gov/pmc/articles/PMC3114256/table/T2/)

CETWRTETTGATGQASSLLSGRLLEQKAASCHNSYIVLCIENSFMTSFSK

>Endostatin peptide fragment I (180?199) (http://www.ncbi.nlm.nih.gov/pmc/articles/PMC3114256/table/T2/)

FLSSRLQDLYSIVRRADRAA

>C16Y (http://www.ncbi.nlm.nih.gov/pmc/articles/PMC3114256/table/T2/)

DFKLFAVYIKYR

>C16S (http://www.ncbi.nlm.nih.gov/pmc/articles/PMC3114256/table/T2/)

DFKLFAVTIKYR

>VEGFderived peptide (http://www.ncbi.nlm.nih.gov/pmc/articles/PMC3114256/table/T3/)

KSVRGKGKGQKRKRKKSRYK

>P144 (http://www.ncbi.nlm.nih.gov/pmc/articles/PMC3114256/table/T3/)

TSLDASIIWAMMQN

>HPRG derived (http://www.ncbi.nlm.nih.gov/pmc/articles/PMC3114256/table/T4/)

HHPHGHHPHGHHPHGHHPHG

>KV11 (http://www.ncbi.nlm.nih.gov/pmc/articles/PMC3114256/table/T4/)

YTMNPRKLFDY

>Fibrinogen derived (http://www.ncbi.nlm.nih.gov/pmc/articles/PMC3114256/table/T4/)

ARPAKAAATQKKVERKAPDA

>PF4 derived (http://www.ncbi.nlm.nih.gov/pmc/articles/PMC3114256/table/T5/)

NGRKISLDLRAPLYKKIIKKLLES

>Chemokinostatin-1 (http://www.ncbi.nlm.nih.gov/pmc/articles/PMC3114256/table/T5/)

NGREACLDPEAPMVQKIVQKMLKG

>Anginex (http://www.ncbi.nlm.nih.gov/pmc/articles/PMC3114256/table/T5/)

ANIKLSVQMKLFKRHLKWKIIVKLNDGRELSLDA

>ABT-510 (http://www.ncbi.nlm.nih.gov/pmc/articles/PMC3114256/table/T6/)

GVDITVIRPNH

>ABT-526 (http://www.ncbi.nlm.nih.gov/pmc/articles/PMC3114256/table/T6/)

GVDITVIRPNH

>Properdistatin (http://www.ncbi.nlm.nih.gov/pmc/articles/PMC3114256/table/T6/)

GPWEPCSVTCSKGTRTRRR

>PEDF-TGAfragment (http://www.ncbi.nlm.nih.gov/pmc/articles/PMC3114256/table/T7/)

TGALVEEEDPF

>PEDF 34-mer fragment (http://www.ncbi.nlm.nih.gov/pmc/articles/PMC3114256/table/T7/)

DPFFKVPVNKLAAVSNFGYDLYRVRSSMSPTTN

>PEDF P18 (http://www.ncbi.nlm.nih.gov/pmc/articles/PMC3114256/table/T7/)

NFGYDLYRVRSSTSPTTN

>SvOrth-2 (http://www.ncbi.nlm.nih.gov/pmc/articles/PMC3114256/table/T7/)

NVLLSPLSVATALSALSLGAEQRTES

>pTnI (http://www.ncbi.nlm.nih.gov/pmc/articles/PMC3114256/table/T8/)

EDMNQKLFDLRGKFKRPPLRRVRMSADAML

>A? (http://www.ncbi.nlm.nih.gov/pmc/articles/PMC3114256/table/T8/)

EVHHQKLVFF

>Spondinstatin-1 (http://www.ncbi.nlm.nih.gov/pmc/articles/PMC2886297/table/T1/)

SEWSDCSVTCGKGMRTRQR

>Cyrostatin (http://www.ncbi.nlm.nih.gov/pmc/articles/PMC2886297/table/T1/)

TSWSQCSKTCGTGISTRV

>Connectostatin (http://www.ncbi.nlm.nih.gov/pmc/articles/PMC2886297/table/T1/)

TEWSACSKTCGMGISTRV

>Nephroblastostatin (http://www.ncbi.nlm.nih.gov/pmc/articles/PMC2886297/table/T1/)

TEWTACSKSCGMGFSTRV

>Wispostatin-2 (http://www.ncbi.nlm.nih.gov/pmc/articles/PMC2886297/table/T1/)

TAWGPCSTTCGLGMATRV

>Wispostatin-3 (http://www.ncbi.nlm.nih.gov/pmc/articles/PMC2886297/table/T1/)

TKWTPCSRTCGMGISNRV

>Netrinstatin-5C (http://www.ncbi.nlm.nih.gov/pmc/articles/PMC2886297/table/T1/)

TEWSVCNSRCGRGYQKRTR

>Netrinstatin-5D (http://www.ncbi.nlm.nih.gov/pmc/articles/PMC2886297/table/T1/)

TEWSACNVRCGRGWQKRSR

>Adamtsostatin-like-4 (http://www.ncbi.nlm.nih.gov/pmc/articles/PMC2886297/table/T1/)

SPWSQCSVRCGRGQRSRQVR

>Fibulostatin-6.1 (http://www.ncbi.nlm.nih.gov/pmc/articles/PMC2886297/table/T1/)

SAWRACSVTCGKGIQKRSR

>Complestatin-C6 (http://www.ncbi.nlm.nih.gov/pmc/articles/PMC2886297/table/T1/)

TQWTSCSKTCNSGTQSRHR

>Adamtsostatin-4 (http://www.ncbi.nlm.nih.gov/pubmed/17531201)

GPWGDCSRTCGGGVQFSSR

>Adamtsostatin-16 (http://www.ncbi.nlm.nih.gov/pubmed/17531201)

SPWSQCTASCGGGVQTR

>Adamtsostatin-18 (http://www.ncbi.nlm.nih.gov/pubmed/17531201)

SKWSECSRTCGGGVKFQER

>Cartilostatin-1 (http://www.ncbi.nlm.nih.gov/pubmed/17531201)

SPWSKCSAACGQTGVQTRTR

>Cartilostatin-2 (http://www.ncbi.nlm.nih.gov/pubmed/17531201)

GPWGPCSGSCGPGRRLRRR

>Fibulostatin-6.2 (http://www.ncbi.nlm.nih.gov/pubmed/17531201)

ASWSACSVSCGGGARQRTR

>Fibulostatin-6.3 (http://www.ncbi.nlm.nih.gov/pubmed/17531201)

QPWGTCSESCGKGTQTRAR

>Papilostatin-1 (http://www.ncbi.nlm.nih.gov/pubmed/17531201)

GPWAPCSASCGGGSQSRS

>Papilostatin-2 (http://www.ncbi.nlm.nih.gov/pubmed/17531201)

SQWSPCSRTCGGGVSFRER

>Properdistatin (http://www.ncbi.nlm.nih.gov/pubmed/17531201)

GPWEPCSVTCSKGTRTRRR

>Scospondistatin (http://www.ncbi.nlm.nih.gov/pubmed/17531201)

GPWEDCSVSCGGGEQLRSR

>Semastatin-5A.1 (http://www.ncbi.nlm.nih.gov/pubmed/17531201)

GPWERCTAQCGGGIQARRR

>Semastatin-5A.2 (http://www.ncbi.nlm.nih.gov/pubmed/17531201)

SPWTKCSATCGGGHYMRTR

>Semastatin-5B (http://www.ncbi.nlm.nih.gov/pubmed/17531201)

TSWSPCSASCGGGHYQRTR

>Thrombostatin cont-1 (http://www.ncbi.nlm.nih.gov/pubmed/17531201)

QPWSQCSATCGDGVRERRR

>Thrombostatin cont-3 (http://www.ncbi.nlm.nih.gov/pubmed/17531201)

SPWSPCSGNCSTGKQQRTR

>Thrombostatin cont-6 (http://www.ncbi.nlm.nih.gov/pubmed/17531201)

WTRCSSSCGRGVSVRSR

>Wispostatin-1 (http://www.ncbi.nlm.nih.gov/pubmed/17531201)

SPWSPCSTSCGLGVSTRI

>MVF-heR-2-266?296(cYc) (CA 2612394 A1)

LHCPALVTYNTDTFESMPNPEGRYTFGASCV

>VeGF-p3(cYc)p3 (http://www.ncbi.nlm.nih.gov/pmc/articles/PMC3494619/)

ITMQGIQGQKIRMIMF

>RI-VeGF-p4(cYc)p4 (http://www.ncbi.nlm.nih.gov/pmc/articles/PMC3494619/)

IMRIKQGQIGQMTI

>Tetrastatin-1 Supporting Information(Karagiannis and Popel 10.1073/pnas.0803241105)

LPVFSTLPFAYCNIHQVCH

>Tetrastatin-2 Supporting Information(Karagiannis and Popel 10.1073/pnas.0803241105)

YCNIHQVCHYAQRNDRSYWL

>Tetrastatin-3 Supporting Information(Karagiannis and Popel 10.1073/pnas.0803241105)

AAPFLECQGRQGTCHFFAN

>Pentastatin-1 Supporting Information(Karagiannis and Popel 10.1073/pnas.0803241105)

LRRFSTMPFMFCNINNVCNF

>Pentastatin-2 Supporting Information(Karagiannis and Popel 10.1073/pnas.0803241105)

FCNINNVCNFASRNDYSYW

>Pentastatin-3 Supporting Information(Karagiannis and Popel 10.1073/pnas.0803241105)

SAPFIECHGRGTCNYYANS

>Hexastatin-1 Supporting Information(Karagiannis and Popel 10.1073/pnas.0803241105)

ATPFIECSGARGTCHYFAN

>Hexastatin-2 Supporting Information(Karagiannis and Popel 10.1073/pnas.0803241105)

YCNINEVCHYARRNDKSYWL

>Hexastatin-3 Supporting Information(Karagiannis and Popel 10.1073/pnas.0803241105)

LPRFSTMPFIYCNINEVCHY

>Adamtsostatin-4 Supporting Information(Karagiannis and Popel 10.1073/pnas.0803241105)

GPWGDCSRTCGGGVQFSSR

>Adamtsostatin-16 Supporting Information(Karagiannis and Popel 10.1073/pnas.0803241105)

SPWSQCTASCGGGVQTR

>Adamtsostatin-18 Supporting Information(Karagiannis and Popel 10.1073/pnas.0803241105)

SKWSECSRTCGGGVKFQER

>Adamtsostatin-like-4 Supporting Information(Karagiannis and Popel 10.1073/pnas.0803241105)

SPWSQCSVRCGRGQRSRQVR

>Cartilostatin-1 Supporting Information(Karagiannis and Popel 10.1073/pnas.0803241105)

SPWSKCSAACGQTGVQTRTR

>Cartilostatin-2 Supporting Information(Karagiannis and Popel 10.1073/pnas.0803241105)

GPWGPCSGSCGPGRRLRRR

>Cyrostatin Supporting Information(Karagiannis and Popel 10.1073/pnas.0803241105)

TSWSQCSKTCGTGISTRV

>Complestatin-C6Supporting Information(Karagiannis and Popel 10.1073/pnas.0803241105)

TQWTSCSKTCNSGTQSRHR

>Connectostatin Supporting Information(Karagiannis and Popel 10.1073/pnas.0803241105)

TEWSACSKTCGMGISTRV

>Fibulostatin-6.1 Supporting Information(Karagiannis and Popel 10.1073/pnas.0803241105)

SAWRACSVTCGKGIQKRSR

>Fibulostatin-6.2 Supporting Information(Karagiannis and Popel 10.1073/pnas.0803241105)

ASWSACSVSCGGGARQRTR

>Fibulostatin-6.3 Supporting Information(Karagiannis and Popel 10.1073/pnas.0803241105)

QPWGTCSESCGKGTQTRAR

>Nephroblastostatin Supporting Information(Karagiannis and Popel 10.1073/pnas.0803241105)

TEWTACSKSCGMGFSTRV

>Netrinstatin-5C Supporting Information(Karagiannis and Popel 10.1073/pnas.0803241105)

TEWSVCNSRCGRGYQKRTR

>Netrinstatin-5D Supporting Information(Karagiannis and Popel 10.1073/pnas.0803241105)

TEWSACNVRCGRGWQKRSR

>Papilostatin-1 Supporting Information(Karagiannis and Popel 10.1073/pnas.0803241105)

GPWAPCSASCGGGSQSRS

>Papilostatin-2 Supporting Information(Karagiannis and Popel 10.1073/pnas.0803241105)

SQWSPCSRTCGGGVSFRER

>Properdistatin Supporting Information(Karagiannis and Popel 10.1073/pnas.0803241105)

GPWEPCSVTCSKGTRTRRR

>Scospondistatin Supporting Information(Karagiannis and Popel 10.1073/pnas.0803241105)

GPWEDCSVSCGGGEQLRSR

>Semastatin-5A.1 Supporting Information(Karagiannis and Popel 10.1073/pnas.0803241105)

GPWERCTAQCGGGIQARRR

>Semastatin-5A.2 Supporting Information(Karagiannis and Popel 10.1073/pnas.0803241105)

SPWTKCSATCGGGHYMRTR

>Semastatin-5B Supporting Information(Karagiannis and Popel 10.1073/pnas.0803241105)

TSWSPCSASCGGGHYQRTR

>Spondinstatin Supporting Information(Karagiannis and Popel 10.1073/pnas.0803241105)

SEWSDCSVTCGKGMRTRQR

>Thrombostatin con-1 Supporting Information(Karagiannis and Popel 10.1073/pnas.0803241105)

QPWSQCSATCGDGVRERRR

>Thrombostatin con-3 Supporting Information(Karagiannis and Popel 10.1073/pnas.0803241105)

SPWSPCSGNCSTGKQQRTR

>Thrombostatin con-6 Supporting Information(Karagiannis and Popel 10.1073/pnas.0803241105)

WTRCSSSCGRGVSVRSR

>Wispostatin-1 Supporting Information(Karagiannis and Popel 10.1073/pnas.0803241105)

SPWSPCSTSCGLGVSTR

>Wispostatin-2 Supporting Information(Karagiannis and Popel 10.1073/pnas.0803241105)

TAWGPCSTTCGLGMATRV

>Wispostatin-3 Supporting Information(Karagiannis and Popel 10.1073/pnas.0803241105)

TKWTPCSRTCGMGISNRV

>Chemokinostatin-1 Supporting Information(Karagiannis and Popel 10.1073/pnas.0803241105)

NGRKACLNPASPIVKKIIEKMLNS

>Chemokinostatin-3 Supporting Information(Karagiannis and Popel 10.1073/pnas.0803241105)

NGKKACLNPASPMVQKIIEKIL

>Chemokinostatin-5 Supporting Information(Karagiannis and Popel 10.1073/pnas.0803241105)

NGKEICLDPEAPFLKKVIQKILD

>Chemokinostatin-6 Supporting Information(Karagiannis and Popel 10.1073/pnas.0803241105)

NGKQVCLDPEAPFLKKVIQKILDS

>Chemokinostatin-7 Supporting Information(Karagiannis and Popel 10.1073/pnas.0803241105)

DGRKICLDPDAPRIKKIVQKKL

>Chemokinostatin-8 Supporting Information(Karagiannis and Popel 10.1073/pnas.0803241105)

DGRELCLDPKENWVQRVVEKFLK

>Growth Hormone-tilted CAA23779(101?114) Supporting Information(Karagiannis and Popel 10.1073/pnas.0803241105)

LLRISLLLIQSWLE

>Kininogen D5 peptide AAB59550(459?492) Supporting Information(Karagiannis and Popel 10.1073/pnas.0803241105)

HGLGHGHEQQHGLGHGHKFKLDDDLEHQGGHVLD

>AAA84914(44?77) Supporting Information(Karagiannis and Popel 10.1073/pnas.0803241105)

DPFFKVPVNKLAAAVSNFGYDLYRVRSSTSPTTN

>AAA84914(98?114) Supporting Information(Karagiannis and Popel 10.1073/pnas.0803241105)

QRTESIIHRALYYDLIS

>AAA84914(36?46)Supporting Information(Karagiannis and Popel 10.1073/pnas.0803241105)

TGALVEEEDPF

>AAI12094(78?101)Supporting Information(Karagiannis and Popel 10.1073/pnas.0803241105)

LRSRGELVAKFLAGEQSPEDYVAE

> CAA38264(101?114)Supporting Information(Karagiannis and Popel 10.1073/pnas.0803241105)

QQMNQKDFLSLIVS

>TIMP2 AAB19474(171?194) Supporting Information(Karagiannis and Popel 10.1073/pnas.0803241105)

ECLWMDWVTEKNINGHQAKFFACI

>TSP1/Mal2 NP_003237(442?460) Supporting Information(Karagiannis and Popel 10.1073/pnas.0803241105)

SPWSSCSVTCGDGVITRIR

>TSP1/Mal3 NP_003237(499?517) Supporting Information(Karagiannis and Popel 10.1073/pnas.0803241105)

SPWDICSVTCGGGVQKRSR

>Tumstatin/T3 AAF72632(69?88) Supporting Information(Karagiannis and Popel 10.1073/pnas.0803241105)

EGLPGPQGPKGFPGLPGLTG

>Tumstatin/T4 AAF72632(84?103) Supporting Information(Karagiannis and Popel 10.1073/pnas.0803241105)

LPGLTGSKGVRGISGLPGFSG

>Flt2-13 A small peptide derived from Flt-1 (VEGFR-1) functions as an angiogenic inhibitor (http://www.ncbi.nlm.nih.gov/pubmed/11311231)

SPNITVTLKKFPL

>sFlt2-13 A small peptide derived from Flt-1 (VEGFR-1) functions as an angiogenic inhibitor(http://www.ncbi.nlm.nih.gov/pubmed/11311231)

LVPLPKIKNSTFT

>Flt2-13T A small peptide derived from Flt-1 (VEGFR-1) functions as an angiogenic inhibitor(http://www.ncbi.nlm.nih.gov/pubmed/11311231)

STNITVTLKKFPL

>Flt2-11 A small peptide derived from Flt-1 (VEGFR-1) functions as an angiogenic inhibitor(http://www.ncbi.nlm.nih.gov/pubmed/11311231)

NITVTLKKFPL

>Flt1-12 A small peptide derived from Flt-1 (VEGFR-1) functions as an angiogenic inhibitor(http://www.ncbi.nlm.nih.gov/pubmed/11311231)

RPFVEMYSEIPE

>FSEN A small peptide derived from Flt-1 (VEGFR-1) functions as an angiogenic inhibitor(http://www.ncbi.nlm.nih.gov/pubmed/11311231)

CQNHHAKHGKVC

>FSEC A small peptide derived from Flt-1 (VEGFR-1) functions as an angiogenic inhibitor(http://www.ncbi.nlm.nih.gov/pubmed/11311231)

CELDENNTPMC

>scFSEN A small peptide derived from Flt-1 (VEGFR-1) functions as an angiogenic inhibitor(http://www.ncbi.nlm.nih.gov/pubmed/11311231)

KCGHKHQCAVHN

>scFSEC A small peptide derived from Flt-1 (VEGFR-1) functions as an angiogenic inhibitor(http://www.ncbi.nlm.nih.gov/pubmed/11311231)

MEPECNLNCTD

>TSP-1 Non-peptidic Thrombospondin-1 Mimics as Fibroblast Growth Factor-2 Inhibitors (http://www.ncbi.nlm.nih.gov/pmc/articles/PMC2838296/#!po=11.3636 )

DDDDDNDKIPDDRDN

>a2-macroglobulin http://www.ncbi.nlm.nih.gov/pmc/articles/PMC1223577/pdf/12755687.pdf

WDLVVVSAGVAEVGV

>pentraxin-3 (PTX3) (http://www.ncbi.nlm.nih.gov/pubmed/15031207)

ESLARPCAPGAPAEARL
