# Supplementary material for: AntiAngioPred: A Server for Prediction of Anti-Angiogenic Peptides
Source: PLoS One. 2015 Sep 3;10(9):e0136990. doi: 10.1371/journal.pone.0136990 (PMC4559406; doi:10.1371/journal.pone.0136990)
Supplement: S6 Table — (DOCX) [file pone.0136990.s008.docx]

**S6 Table. Propensities of amino acids in anti-angiogenic peptides calculated using Swiss-Prot as reference dataset.**

| **Amino Acid** | **Propensity** |
| --- | --- |
| **A** | 0.38 |
| **C** | 0.76 |
| **D** | 0.47 |
| **E** | 0.41 |
| **F** | 0.43 |
| **G** | 0.53 |
| **H** | 0.58 |
| **I** | 0.43 |
| **K** | 0.48 |
| **L** | 0.41 |
| **M** | 0.42 |
| **N** | 0.48 |
| **P** | 0.59 |
| **Q** | 0.51 |
| **R** | 0.60 |
| **S** | 0.60 |
| **T** | 0.55 |
| **V** | 0.40 |
| **W** | 0.69 |
| **Y** | 0.43 |
