# Supplementary material for: AntiAngioPred: A Server for Prediction of Anti-Angiogenic Peptides
Source: PLoS One. 2015 Sep 3;10(9):e0136990. doi: 10.1371/journal.pone.0136990 (PMC4559406; doi:10.1371/journal.pone.0136990)
Supplement: S7 Table — N1 represents the first residue and C10 represents the last residue. The propensities were calculated using Swiss-Prot as reference dataset. (DOCX) [file pone.0136990.s009.docx]

**S7 Table. Positional propensity of amino acids in first and last 10 residues of N- and C- terminus of anti-angiogenic peptides. N1 represents the first residue and C10 represents the last residue. The propensities were calculated using Swiss-Prot as reference dataset.**

| **AA** | **N1** | **N2** | **N3** | **N4** | **N5** | **N6** | **N7** | **N8** | **N9** | **N10** | **C1** | **C2** | **C3** | **C4** | **C5** | **C6** | **C7** | **C8** | **C9** | **C10** |
| --- | --- | --- | --- | --- | --- | --- | --- | --- | --- | --- | --- | --- | --- | --- | --- | --- | --- | --- | --- | --- |
| **A** | 0.31 | 0.31 | 0.15 | 0.26 | 0.55 | 0.38 | 0.31 | 0.49 | 0.34 | 0.41 | 0.41 | 0.34 | 0.41 | 0.38 | 0.21 | 0.49 | 0.49 | 0.38 | 0.34 | 0.38 |
| **C** | 0.72 | 0.67 | 0.51 | 0.51 | 0.67 | 0.95 | 0.75 | 0.75 | 0.51 | 0.93 | 0.91 | 0.75 | 0.72 | 0.78 | 0.75 | 0.51 | 0.51 | 0.61 | 0.34 | 0.51 |
| **D** | 0.61 | 0.46 | 0.30 | 0.58 | 0.50 | 0.36 | 0.41 | 0.56 | 0.46 | 0.30 | 0.36 | 0.46 | 0.50 | 0.22 | 0.53 | 0.58 | 0.61 | 0.12 | 0.61 | 0.36 |
| **E** | 0.40 | 0.50 | 0.25 | 0.59 | 0.31 | 0.61 | 0.31 | 0.36 | 0.18 | 0.36 | 0.44 | 0.36 | 0.40 | 0.31 | 0.53 | 0.47 | 0.40 | 0.36 | 0.47 | 0.31 |
| **F** | 0.37 | 0.37 | 0.57 | 0.66 | 0.16 | 0.28 | 0.00 | 0.49 | 0.43 | 0.37 | 0.49 | 0.28 | 0.37 | 0.53 | 0.43 | 0.37 | 0.43 | 0.61 | 0.28 | 0.49 |
| **G** | 0.51 | 0.57 | 0.38 | 0.45 | 0.59 | 0.38 | 0.34 | 0.55 | 0.55 | 0.34 | 0.53 | 0.69 | 0.70 | 0.74 | 0.55 | 0.29 | 0.38 | 0.29 | 0.29 | 0.55 |
| **H** | 0.67 | 0.50 | 0.40 | 0.57 | 0.62 | 0.57 | 0.50 | 0.62 | 0.57 | 0.50 | 0.57 | 0.62 | 0.57 | 0.57 | 0.62 | 0.62 | 0.62 | 0.40 | 0.67 | 0.40 |
| **I** | 0.49 | 0.33 | 0.38 | 0.52 | 0.52 | 0.27 | 0.46 | 0.27 | 0.52 | 0.42 | 0.27 | 0.42 | 0.49 | 0.52 | 0.52 | 0.46 | 0.33 | 0.49 | 0.27 | 0.38 |
| **K** | 0.42 | 0.49 | 0.32 | 0.52 | 0.49 | 0.52 | 0.38 | 0.55 | 0.19 | 0.59 | 0.61 | 0.52 | 0.55 | 0.26 | 0.46 | 0.63 | 0.46 | 0.46 | 0.49 | 0.52 |
| **L** | 0.38 | 0.32 | 0.46 | 0.28 | 0.38 | 0.35 | 0.58 | 0.44 | 0.38 | 0.44 | 0.44 | 0.46 | 0.35 | 0.41 | 0.44 | 0.19 | 0.46 | 0.46 | 0.24 | 0.46 |
| **M** | 0.54 | 0.47 | 0.54 | 0.00 | 0.00 | 0.37 | 0.37 | 0.37 | 0.37 | 0.54 | 0.00 | 0.37 | 0.00 | 0.54 | 0.54 | 0.37 | 0.64 | 0.37 | 0.64 | 0.00 |
| **N** | 0.49 | 0.60 | 0.43 | 0.49 | 0.36 | 0.43 | 0.49 | 0.43 | 0.43 | 0.49 | 0.43 | 0.49 | 0.43 | 0.53 | 0.53 | 0.43 | 0.36 | 0.27 | 0.53 | 0.60 |
| **P** | 0.25 | 0.79 | 0.74 | 0.50 | 0.66 | 0.50 | 0.50 | 0.45 | 0.66 | 0.50 | 0.33 | 0.45 | 0.50 | 0.57 | 0.54 | 0.54 | 0.50 | 0.62 | 0.54 | 0.45 |
| **Q** | 0.54 | 0.44 | 0.16 | 0.37 | 0.61 | 0.16 | 0.50 | 0.44 | 0.58 | 0.58 | 0.54 | 0.66 | 0.28 | 0.50 | 0.50 | 0.73 | 0.64 | 0.50 | 0.37 | 0.16 |
| **R** | 0.51 | 0.58 | 0.62 | 0.34 | 0.62 | 0.43 | 0.58 | 0.54 | 0.61 | 0.51 | 0.54 | 0.54 | 0.61 | 0.43 | 0.47 | 0.70 | 0.47 | 0.77 | 0.73 | 0.79 |
| **S** | 0.68 | 0.52 | 0.61 | 0.74 | 0.57 | 0.52 | 0.78 | 0.54 | 0.59 | 0.54 | 0.52 | 0.61 | 0.61 | 0.37 | 0.42 | 0.42 | 0.67 | 0.54 | 0.54 | 0.63 |
| **T** | 0.68 | 0.53 | 0.41 | 0.63 | 0.50 | 0.46 | 0.50 | 0.50 | 0.73 | 0.41 | 0.63 | 0.46 | 0.50 | 0.65 | 0.46 | 0.22 | 0.53 | 0.56 | 0.71 | 0.46 |
| **V** | 0.24 | 0.39 | 0.24 | 0.34 | 0.34 | 0.39 | 0.42 | 0.61 | 0.34 | 0.39 | 0.46 | 0.24 | 0.39 | 0.46 | 0.56 | 0.39 | 0.34 | 0.39 | 0.24 | 0.46 |
| **W** | 0.58 | 0.00 | 0.95 | 0.58 | 0.58 | 0.58 | 0.41 | 0.00 | 0.74 | 0.00 | 0.00 | 0.41 | 0.00 | 0.00 | 0.68 | 0.58 | 0.41 | 0.74 | 0.68 | 0.58 |
| **Y** | 0.51 | 0.34 | 0.34 | 0.00 | 0.00 | 0.44 | 0.51 | 0.44 | 0.44 | 0.34 | 0.00 | 0.44 | 0.51 | 0.20 | 0.56 | 0.64 | 0.61 | 0.56 | 0.61 | 0.20 |
