# Supplementary material for: AntiAngioPred: A Server for Prediction of Anti-Angiogenic Peptides
Source: PLoS One. 2015 Sep 3;10(9):e0136990. doi: 10.1371/journal.pone.0136990 (PMC4559406; doi:10.1371/journal.pone.0136990)
Supplement: S8 Table — (DOCX) [file pone.0136990.s010.docx]

**S8 Table. List of motifs extracted by MERCI software.**

| **MOTIF** | **Frequency_in_positive_dataset** | **Frequency_in_negative_dataset** | **Propensity** |
| --- | --- | --- | --- |
| CG-G | 23 | 0 | 1 |
| TC | 19 | 0 | 1 |
| SC | 17 | 0 | 1 |
| SP-S | 16 | 0 | 1 |
| W-S-C | 15 | 0 | 1 |
| WS-C | 14 | 0 | 1 |
| S-T-C | 14 | 0 | 1 |
| S-C-S | 14 | 0 | 1 |
| CS-T | 14 | 0 | 1 |
| C-S-T | 14 | 0 | 1 |
| T-C | 24 | 1 | 0.96 |
| S-C | 27 | 1 | 0.96 |
| C-G-G | 24 | 1 | 0.96 |
| TR | 20 | 1 | 0.95 |
| S-T-G | 16 | 1 | 0.94 |
| S-P-S | 17 | 1 | 0.94 |
| SP | 28 | 2 | 0.93 |
| RT | 20 | 2 | 0.91 |
| P-W | 18 | 2 | 0.90 |
| P-C | 18 | 2 | 0.90 |
| C-N | 18 | 2 | 0.90 |
| CG | 26 | 3 | 0.90 |
| PW | 16 | 2 | 0.89 |
| E-C | 16 | 2 | 0.89 |
| S-W | 23 | 3 | 0.88 |
| Q-R-R | 14 | 2 | 0.88 |
| CS | 26 | 4 | 0.87 |
| C-G | 31 | 6 | 0.84 |
| C-S | 30 | 6 | 0.83 |
| S-G-G | 14 | 3 | 0.82 |
| C-L | 18 | 4 | 0.82 |
| WS | 16 | 4 | 0.80 |
| Q-R | 27 | 7 | 0.79 |
| W-S | 18 | 5 | 0.78 |
| R-C | 14 | 4 | 0.78 |
| T-R | 24 | 7 | 0.77 |
| S-P | 33 | 10 | 0.77 |
| A-C | 20 | 6 | 0.77 |
| P-S | 29 | 9 | 0.76 |
| AS | 19 | 6 | 0.76 |
| C-A | 15 | 5 | 0.75 |
| R-T | 29 | 10 | 0.74 |
| QR | 14 | 5 | 0.74 |
| G-Q | 23 | 8 | 0.74 |
| R-S | 24 | 9 | 0.73 |
| KR | 19 | 7 | 0.73 |
| TG | 14 | 6 | 0.70 |
| S-T | 33 | 14 | 0.70 |
| S-S | 37 | 16 | 0.70 |
| R-Q | 14 | 6 | 0.70 |
| QK | 16 | 7 | 0.70 |
| V-C | 18 | 8 | 0.69 |
| T-G | 29 | 13 | 0.69 |
| SR | 20 | 9 | 0.69 |
| R-R | 37 | 17 | 0.69 |
| S-G | 30 | 14 | 0.68 |
| RG | 21 | 10 | 0.68 |
| K-R | 22 | 11 | 0.67 |
| A-S | 26 | 13 | 0.67 |
| S-R | 26 | 14 | 0.65 |
| S-K | 17 | 9 | 0.65 |
| P-F | 17 | 9 | 0.65 |
| I-N | 16 | 9 | 0.64 |
| ST | 17 | 10 | 0.63 |
| E-S | 24 | 14 | 0.63 |
| T-T | 15 | 9 | 0.62 |
| Q-K | 18 | 11 | 0.62 |
| P-R | 15 | 9 | 0.62 |
| P-E | 20 | 12 | 0.62 |
| G-R | 32 | 20 | 0.62 |
| D-P | 16 | 10 | 0.62 |
| SS | 18 | 12 | 0.60 |
| G-G | 34 | 23 | 0.60 |
| V-S | 19 | 13 | 0.59 |
| SV | 19 | 14 | 0.58 |
| SG | 15 | 11 | 0.58 |
| P-K | 14 | 10 | 0.58 |
| K-F | 15 | 11 | 0.58 |
| S-A | 23 | 17 | 0.57 |
| K-S | 21 | 16 | 0.57 |
| GG | 16 | 12 | 0.57 |
| D-R | 16 | 12 | 0.57 |
| T-S | 20 | 16 | 0.56 |
| GR | 20 | 16 | 0.56 |
| A-R | 22 | 17 | 0.56 |
| SA | 16 | 13 | 0.55 |
| R-K | 18 | 15 | 0.55 |
| R-G | 24 | 20 | 0.55 |
| L-S | 24 | 20 | 0.55 |
| K-K | 18 | 15 | 0.55 |
| F-L | 17 | 14 | 0.55 |
| R-P | 21 | 18 | 0.54 |
| D-S | 19 | 16 | 0.54 |
| T-E | 17 | 15 | 0.53 |
| P-G | 18 | 16 | 0.53 |
| K-I | 19 | 17 | 0.53 |
| A-P | 20 | 18 | 0.53 |
| Q-S | 14 | 13 | 0.52 |
| N-G | 15 | 14 | 0.52 |
| K-G | 16 | 15 | 0.52 |
| I-K | 14 | 13 | 0.52 |
| GV | 14 | 13 | 0.52 |
| G-P | 17 | 16 | 0.52 |
| GK | 16 | 15 | 0.52 |
| S-V | 23 | 22 | 0.51 |
| S-L | 25 | 24 | 0.51 |
| P-L | 20 | 19 | 0.51 |
| G-K | 27 | 26 | 0.51 |
| V-T | 18 | 18 | 0.50 |
| SL | 17 | 17 | 0.50 |
| S-D | 16 | 16 | 0.50 |
| R-V | 16 | 16 | 0.50 |
| R-I | 18 | 18 | 0.50 |
| R-A | 17 | 17 | 0.50 |
| I-S | 16 | 16 | 0.50 |
| I-R | 15 | 15 | 0.50 |
| G-T | 17 | 17 | 0.50 |
| V-R | 18 | 19 | 0.49 |
| T-P | 18 | 19 | 0.49 |
| I-T | 18 | 19 | 0.49 |
| P-D | 15 | 16 | 0.48 |
| LD | 14 | 15 | 0.48 |
| P-A | 14 | 16 | 0.47 |
| L-P | 14 | 16 | 0.47 |
| G-S | 22 | 25 | 0.47 |
| T-A | 17 | 20 | 0.46 |
| R-E | 16 | 19 | 0.46 |
| L-V | 18 | 21 | 0.46 |
| S-I | 14 | 17 | 0.45 |
| R-L | 20 | 25 | 0.44 |
| K-L | 18 | 23 | 0.44 |
| L-D | 22 | 29 | 0.43 |
| RL | 14 | 19 | 0.42 |
| L-G | 20 | 28 | 0.42 |
| V-L | 17 | 26 | 0.40 |
| L-R | 19 | 29 | 0.40 |
| L-K | 18 | 27 | 0.40 |
| L-A | 20 | 30 | 0.40 |
| G-V | 17 | 26 | 0.40 |
| E-L | 19 | 29 | 0.40 |
| L-E | 16 | 26 | 0.38 |
| I-L | 15 | 24 | 0.38 |
| G-A | 19 | 31 | 0.38 |
| D-L | 14 | 23 | 0.38 |
| L-T | 14 | 25 | 0.36 |
| A-G | 14 | 25 | 0.36 |
| A-A | 17 | 30 | 0.36 |
| L-L | 15 | 30 | 0.33 |
| I-V | 14 | 30 | 0.32 |
| G-L | 15 | 33 | 0.31 |
| A-L | 18 | 41 | 0.31 |
